# Mucosal Barrier and Th2 Immune Responses Are Enhanced by Dietary Inulin in Pigs Infected With *Trichuris suis*

**DOI:** 10.3389/fimmu.2018.02557

**Published:** 2018-11-09

**Authors:** Laura J. Myhill, Sophie Stolzenbach, Tina V. A. Hansen, Kerstin Skovgaard, C. Rune Stensvold, Lee O'Brien Andersen, Peter Nejsum, Helena Mejer, Stig M. Thamsborg, Andrew R. Williams

**Affiliations:** ^1^Department of Veterinary and Animal Sciences, Faculty of Health and Medical Sciences, University of Copenhagen, Copenhagen, Denmark; ^2^Department of Biotechnology and Biomedicine, Technical University of Denmark, Kongens Lyngby, Denmark; ^3^Department of Bacteria, Parasites and Fungi, Statens Serum Institut, Copenhagen, Denmark; ^4^Department of Clinical Medicine, Aarhus University, Aarhus, Denmark

**Keywords:** prebiotics, mucosal immunity, gut microbiota, helminth infection, porcine models

## Abstract

Diet composition may play a crucial role in shaping host immune responses and commensal gut microbiota populations. Bioactive dietary components, such as inulin, have been extensively studied for their bioactive properties, particularly in modulating gut immune function and reducing inflammation. It has been shown that colonization with gastrointestinal parasitic worms (helminths) may alleviate chronic inflammation through promotion of T-helper cell type (Th) 2 and T-regulatory immune responses and alterations in the gut microbiome. In this study, we investigated if dietary inulin could modulate mucosal immune function in pigs during colonization with the porcine whipworm *Trichuris suis*. *T. suis* infection induced a typical Th2-biased immune response characterized by transcriptional changes in Th2- and barrier function-related genes, accompanied by intestinal remodeling through increased epithelial goblet and tuft cell proliferation. We observed that inulin also up-regulated Th2-related immune genes (*IL13, IL5*), and suppressed Th1-related pro-inflammatory genes (*IFNG, IL1A, IL8*) in the colon. Notably, inulin augmented the *T. suis*-induced responses with increased transcription of key Th2 and mucosal barrier genes (e.g., *IL13, TFF3*), and synergistically suppressed pro-inflammatory genes, such as *IFNG* and *CXCL9*. 16S rRNA sequencing of proximal colon digesta samples revealed that inulin supplementation reduced the abundance of bacterial phyla linked to inflammation, such as Proteobacteria and Firmicutes, and simultaneously increased Actinobacteria and Bacteroidetes. Interestingly, pigs treated with both inulin and *T. suis* displayed the highest Bacteroidetes: Firmicutes ratio and the lowest gut pH, suggesting an interaction of diet and helminth infection that stimulates the growth of beneficial bacterial species. Overall, our data demonstrate that *T. suis* infection and inulin co-operatively enhance anti-inflammatory immune responses, which is potentially mediated by changes in microbiota composition. Our results highlight the intricate interactions between diet, immune function and microbiota composition in a porcine helminth infection model. This porcine model should facilitate further investigations into the use of bioactive diets as immunomodulatory mediators against inflammatory conditions, and how diet and parasites may influence gut health.

## Introduction

Gut health and mucosal immune function may be considerably influenced by diet, and there is increasing interest in the role of bioactive dietary components as functional food ingredients that may modulate chronic inflammation and immune responses to gastrointestinal microbes. Functional food relates to the ability of a foodstuff to deliver not only nutrients and energy when consumed as part of a normal diet, but also to contribute to protective effects against disease and improve overall health and well-being (1).

The gastrointestinal tract is one of the largest interfaces of the body exposed to external stimuli, and so is highly immunologically active with a myriad of immune cells residing in the mucosa and gut-associated lymphoid tissue (GALT). The functional activity of these mucosal immune cells can be regulated by a variety of different commensal microbes and their products, or external stimuli, such as dietary compounds and gastrointestinal pathogens. Dietary-mediated immunomodulation can occur (i) indirectly through prebiotic modification of the commensal gut microbiota, whereby microbial-derived products, such as short-chain fatty acids (SCFAs) can induce tolerogenic activity in dendritic cells (DCs) and T-cells (2, 3), as well as enhance mucus production and barrier function (4, 5), or (ii) by direct interaction of dietary compounds with the epithelial cell barrier and intestinal immune cells (6). Thus, bioactive dietary components with either prebiotic or direct stimulatory effects may play a major role in modulating mucosal immune function.

One well-studied group of bioactive compounds are inulin-type fructans, which encompass inulin, oligofructose, fructooligosaccharides, among others (7), and are comprised of β2 → 1-fructans with varying chain length (degree of polymerization [DP]) ranging from 2 to 60 (8). This composition resists digestion in the small intestine, and so passes through to the colon for fermentation by commensal microbiota. Fermentation results in the production of SCFAs (butyrate, acetate and propionate), subsequently lowering gut pH and further promoting growth of beneficial bacterial species, such as Lactobacilli and Bifidobacteria (9)—which are considered some of the main contributors to the immunomodulatory effect of fructans. Bifidobacteria are also butyrate producers, and in turn provide a primary energy source for intestinal epithelial cells (IECs), and indirectly promote intestinal barrier function by enhancing tight junction assembly *in vitro* (10). Immune cells present in the epithelial cell barrier can also be directly stimulated by dietary fructans. In particular, DCs are not only activated indirectly via products of commensal Bifidobacteria (11, 12), but also via interactions with fiber-stimulated IECs (13), which can result in enhanced anti-inflammatory IL-10 secretion. It is likely that both direct and indirect mechanisms contribute to the immunomodulatory effects of fructans, as suggested by Fransen et al. (14), who observed both microbiota-dependent and independent effects of β2 → 1-fructans of varying chain lengths.

This complex interaction between diet and the mucosal immune system may profoundly modulate immune responses elicited by gastrointestinal bacteria and parasites. For example, experimental animal studies have reported that inulin-like fructan supplementation can modulate responses to *Salmonella typhimurium* and *Listeria* infection in mice (15, 16). Parasitic helminths are one of the most highly prevalent and widespread infectious agents in both humans and animals worldwide, and, unlike most bacteria and viruses, helminths normally induce polarized T-helper cell type (Th) 2 mucosal immune responses, making them an excellent model for exploring the influence of diet on development of Th2 immune function. The development and maintenance of Th2 immunity in the gut has received increased attention during recent years as, in addition to being relevant for immunity to parasites, it may contribute to reductions in chronic inflammation, obesity and other metabolic disorders (17, 18).

The porcine whipworm *Trichuris suis* is a common parasite of pigs and is closely related to the human whipworm *T. trichiura*, which is found most commonly in developing countries. Similar to most helminths, *T. suis* induces a Th2-biased immune response, characterized by a strong but transient up-regulation of Th2-related genes and a corresponding suppression of pro-inflammatory Th1 responses 3–5 weeks after primary infection. Subsequently, the worms are removed from the gut in a self-cure reaction beginning around 8–9 weeks post-infection (19, 20). Due to these putative anti-inflammatory effects, controlled infection with *T. suis*, or other Th2-inducing helminths, such as the hookworm *Necator americanus*, has been explored as a novel treatment option against autoimmune diseases and inflammatory disorders in humans (21, 22). Proliferation of regulatory T-cells (Treg) and associated dampening of Th1 responses are considered the main anti-inflammatory effector mechanisms observed upon treatment, and an involvement of the gut microbiota has also been proposed (23, 24).

As it is clear that both inulin and *T. suis* colonization can have profound effects on host immune status, and possibly on gut microbiome composition and function, we explored here whether dietary supplementation could effectively augment the acquisition of Th2-immune responses and dampening of pro-inflammatory responses in the colonic mucosa during establishment of *T. suis* in pigs. Specifically, we investigated the effects of feeding purified long-chain inulin to *T. suis*-infected pigs on systemic immune responses, transcription of mucosal barrier genes, and gut microbiota composition in the colon, with the aim of understanding how bioactive diets may modulate a naturally-induced mucosal Th2-biased response.

## Materials and methods

### Animals

Thirty-four crossbred Yorkshire-Landrace pigs (16 castrated males, 18 females) were purchased from a certified specific pathogen-free (SPF) farm with no history of helminth infection. The pigs were ~8 weeks of age and weighed 20.6 ± 2.1 kg [mean ± standard deviation (SD)] on arrival. For the duration of the study, pigs were housed on solid concrete floored pens with feed provided twice daily and water provided *ad libitum* throughout the study. Animal welfare checks were performed daily, and body weight and fecal consistency was recorded weekly. All experimentation was conducted in line with the Danish Animal Experimentation Inspectorate (License number 2015-15-0201-00760), and approved by the Experimental Animal Unit, University of Copenhagen according to FELASA guidelines and recommendations. All animals were parasite free as confirmed by McMaster fecal egg count and serology at arrival.

### Experimental design

The study was designed as 2-factorial (diet and infection; Supplementary Material). Pigs were stratified on the basis of sex and bodyweight and randomly allocated into four treatment groups of eight or nine pigs, with each group housed in two pens of 4–5 pigs. Two groups, totalling 17 pigs, were fed a commercial pre-mixed diet based on ground barley and wheat (“control diet”), whilst the remaining 17 pigs were fed the “experimental diet” containing 10% (w/w) long-chain purified chicory inulin-enriched (Orafti®HP, Beneo, Belgium) diet balanced to the control diet for energy and percentage crude protein intake (Table S1). After 2 weeks of acclimatization to the diet, half the pigs in each dietary group were inoculated with 10,000 embryonated *T. suis* eggs by oral gavage, a dose which has been shown to stimulate a strong Th2 response 3–5 weeks post-infection (p.i.) (20, 25). During the course of the study three pigs were euthanized due to cause's unrelated to experimental treatment. All 31 remaining pigs were sacrificed by stunning with captive bolt followed by exsanguination, and necropsied at day 28 p.i.

Throughout the study period individual blood and fecal samples were collected at day 0, 7, 14, 21, and 28 days p.i. The fecal samples were scored following a 5-point scale in order to monitor fecal consistency, and upon collection immediately cooled to ~4°C before transfer to −80°C for storage prior to microbiota analysis. Blood samples were processed to collect serum and isolate peripheral blood mononuclear cells (PBMCs; see below).

At necropsy, ~5 cm of proximal colon located 20 cm distal to the caecum, was dissected along with ileo-caecal lymph nodes (CLN). Each tissue was lightly washed with phosphate buffered saline (PBS; Sigma-Aldrich, Denmark) and snap-frozen in liquid nitrogen or stored in RNAlater (Invitrogen®, Thermo Fisher Scientific, Denmark). Additional CLN tissue was stored in RPMI 1640 media (Life Technologies, Denmark) supplemented with 10% fetal calf serum (Sigma-Aldrich) and 100 U/mL penicillin + 100 μg/mL streptomycin (complete media) and stored on ice until processing. Full thickness tissue sections were dissected from both the caecum and proximal colon for histological analyses; one tissue sample was stored in Carnoy's fixative (VWR, Denmark), whilst the other was stored in 4% paraformaldehyde.

### Digesta sampling for physiological analyses and worm isolation

Fresh intestinal digesta samples were removed from the ileum (10 cm proximal to the ileo-caecal junction), caecum (blind end), proximal (20 cm from the caecum) and distal (midway between caecum and rectum) colon of each pig for immediate pH measurement (Metrohm Nordic Aps, Denmark). Additional samples were snap-frozen in liquid nitrogen and stored at −80°C for microbiota and SCFA analysis. SCFA concentrations were measured by gas chromatography as previously described in Williams et al. (26). For *T. suis* isolation, all luminal contents were retained from the caecum and proximal colon, and combined with mucosal scrapings to recover attached worms. Luminal and mucosal contents were then washed with tap water, and worms isolated on a 212 μM mesh. A 10% sub-sample was then fixed with iodine (Glostrup Apotek, Denmark) and used as a representative sample for worm enumeration.

### Histology and immunohistochemistry

All full-thickness proximal colon tissue sections were paraffin-embedded, sectioned, and mounted on glass slides. Paraformaldehyde-fixed tissue sections were stained with Luna's stain for eosinophil enumeration, whilst sections fixed in Carnoy's fixative were stained with Toluidine Blue to visualize mast cells. Both cell types were enumerated as described in Williams et al. (26).

Additional paraformaldehyde-fixed sections were sectioned and incubated with 1) doublecortin-like kinase 1 (DCLK1) antibody (R&D Systems, UK), and counter stained with periodic-acid Schiff (PAS) stain enabling tuft cell and goblet cell visualization.

### Enzyme-linked immunosorbent assay

Serum obtained from whole blood by centrifugation was used to quantify antibodies produced specifically in response to adult *T. suis* excretory/secretory (E/S) antigen by enzyme-linked immunosorbent assay (ELISA) as described in full by Dige et al. (27). Primary monoclonal antibodies used to detect bound antibody were porcine immunoglobulin (Ig) A (K61-1B4; Serotec, UK), and porcine IgG1 (K139-3C8, Serotec) followed by anti-mouse IgG conjugated to horseradish peroxidase (HRP; Bio-Rad, Germany). ELISA data are represented as ELISA units (EU) which involved assigning an arbitrary concentration value of 1 × 10^6^ EU to the top standard concentration from a positive control standard dilution series. All sample absorbance data were then converted to EU.

### PBMC isolation and flow cytometry

PBMCs were isolated from heparinised whole blood using Histopaque®-1077 (Sigma-Aldrich) and centrifugation. PBMCs were then incubated on ice for 20 min with the following antibodies: FITC-conjugated mouse anti-pig CD3ε (clone BB23-8E6-8C8; BD Biosciences, Denmark), PerCP-CY™5.5-conjugated mouse anti-pig CD4 (74-12-4; BD Biosciences), Alexa Fluor® 647-conjugated mouse anti-pig CD8a (76-2-11; BD Biosciences) for phenotyping of T cell subsets; or FITC-conjugated mouse anti-pig CD14 (MIL2; Bio-Rad) for monocytes. Appropriate isotype controls were also included. For intracellular staining, the cells were prepared using a fixation/-permeabilization solution kit (BD Cytofix/Cytoperm kit; BD Biosciences), and then incubated on ice for 20 min with Alexa Fluor® 647-conjugated mouse anti-human CD79a (HM57; Bio-Rad) for detection of B cells. Samples were processed on a BD Accuri C6 flow cytometer (BD Biosciences), and the data acquired using Accuri CFlow Plus software (Accuri® Cytometers Inc., MI, USA).

### *In vitro* cell stimulation

Single cell suspensions were prepared from CLN on ice by straining through a 70 μM cell strainer. Cells were then washed, counted and either analyzed by flow cytometry (see above) or suspended at 2.5 × 10^6^ cells/mL in complete media with either 10 μg/mL phytohaemagglutinin (PHA) (Sigma-Aldrich), 20 μg/mL *T. suis* E/S or media only (control). After 48 h incubation at 37°C, culture media was harvested and frozen at −20°C. IFN-γ and IL-10 concentrations were measured using ELISA kits according to manufacturer's instructions (R&D Systems).

### Quantitative real-time PCR

RNA was extracted from ~30 mg proximal colon tissue using a commercial kit (miRNeasy® Mini Kit, Qiagen, CA, USA) in accordance to the manufacturer's guidelines. Briefly, tissue stored in RNAlater was homogenized in QIAzol lysis buffer using a gentleMACS™ dissociator (Miltenyi Biotec, Germany). Purity and concentration of total RNA were then measured using a NanoDrop ND-1000 spectrophotometer (NanoDrop Technologies, DE, USA), and RNA integrity was measured using an Agilent 2100 Bioanalyzer (Agilent, CA, USA). cDNA synthesis and pre-amplification was carried out as described by Williams et al. (26). Expression levels of a panel of 91 genes of interest (GOI), including key Th1/Th2/Treg immune response-related genes and mucosal barrier function-related genes, were examined on a BioMark HD Reader (Fluidigm, CA, USA) under the following conditions: 50°C for 2 min, 95°C for 10 min followed by 35 cycles at 95°C for 15 s and 60°C for 1 min. After data pre-processing, 56 GOI were statistically analyzed (Table S2). Normalization and data pre-processing was carried out as described in Skovgaard et al. (28), with respect to three reference housekeeping genes (Table S2).

### Amplicon sequencing for microbiota analysis

DNA was extracted from proximal colon digesta (*n* = 31) using Mo Bio PowerSoil Kit (Mo Bio Laboratories, CA, USA) according to manufacturer's guidelines. DNA concentration was measured on a NanoDrop ND-1000 Spectrophotometer. Subsequently, the V3-V4 region of the 16S rRNA gene was amplified using the universal forward primer 341F3 (5′-ACTCCTAYGGGRBGCASCAG-3′) and reverse primer 806R5 (5′-AGCGTGGACTACNNGGGTATCTAAT-3′) (pmid15696537). Inserts of 0–19 nucleotides were incorporated before the 5′-end of each primer to enhance complexity during the sequencing by forcing a 1:1:1:1 ratio of each nucleotide for the first 20 sequencing cycles, in line with the principles of phased amplicon sequencing (pmid26084274). The library preparation was done by an initial polymerase chain reaction (PCR) consisting of 12.5 μL Extract-N-Amp™ PCR ReadyMix™ (Sigma-Aldrich, CA, USA), 1 μL of each primer (10 μM), 2 μL of genomic DNA (~10 ng/μL) and nuclease-free water to a total volume of 25 μL, which was run on a Life ECO thermocycler (Bioer, Hangzhou, China). Standard PCR cycling was applied with an initial denaturation at 94°C for 4 min, followed by 20 cycles of 94°C for 30 s, 60°C for 30 s and 72°C for 30 s, with a final elongation at 72°C for 5 min. The PCR products from the initial PCR step were used as templates in the second PCR, which incorporated adapters (A or B), a sequencing primer site (forward or reverse), and indexes (i7 or i5), respectively of forward and reverse reads. Two microlitres of the initial PCR products were used with 12.5 μL Extract-N-Amp™ PCR ReadyMix™, 1 μL of corresponding adapter/index primers each, and nuclease-free water to a total volume of 25 μL. The PCR cycling was identical to the initial PCR. The amplified fragments were quantified using a Qubit Fluorometer (Invitrogen, CA, USA), and pooled in equimolar amounts. The pooled library was purified using size-exclusion Agencourt AMPure XP beads (Beckman-Coulter, CA, USA), prior to Illumina-based sequencing on a MiSeq (Illumina Inc., CA, USA) at Statens Serum Institut (Denmark).

### Bioinformatics and statistical analysis of microbiota

Raw microbiota sequencing data analysis was performed using the BIONmeta package (Danish Genome Institute, Denmark; Figure S2). In brief, the workflow consisted of de-multiplexing sequences according to primers and barcodes, followed by removal of primer remnants from both ends, and regions with a base quality below 99%. Sequences were pair mate joined, and all sequences shorter than 250 base pairs (bp) and with a base quality of < 99% in 95% of the full sequence were removed. The remaining sequences were chimera-checked with a sequence similarity of 96%. The non-chimera sequences were merged while preserving original read count and matched against the Ribosomal Database Project (RDP) subset matching the targeted amplicon. Sequences were divided into 8-mers and required a minimum oligo similarity of 96% to be mapped to the RDP taxonomy, and abundance tables were generated for all levels from phylum to species normalized to 100,000 reads per sample. Data analysis was performed using MacQIIME software (v1.9.1). The relative distribution of registered phyla and families were calculated based on the normalized abundance table, and summarized at phylum and family level abundance tables.

Principal coordinates analysis (PCoA) plots were generated using the Jack-knifed Beta Diversity workflow based on 10 distance matrices using 10 subsampled abundance tables (QIIME v1.9.1). The number of sequences taken for each jack-knifed subset was set to 90% of the sequence number within all samples (100,000 reads per sample). Bray-Curtis and Sorensen-Dice distance matrices were based on rarefied (90,000 reads per sample) abundance tables, and tested for separation between groups with analysis of similarities (ANOSIM). The alpha diversity measures for an observed species (96% oligo similarity) were computed for rarefied abundance table (90,000 reads per sample) using the alpha rarefaction workflow (QIIME v1.9.1). The datasets generated for this study can be found in the European Nucleotide Archive (ENA), under the accession number: PRJEB29079.

### Statistical analysis

All data (including microbiota phyla and family data) were checked for normality using Shapiro-Wilk tests correlation coefficient using GraphPad Prism 7 (GraphPad Software Inc., CA, USA). All data that were not normally distributed were log- or square root-transformed to obtain normal distribution, if possible. Data were analyzed using a mixed linear model (MLM) using IBM SPSS Statistics 24. The model included infection status, diet and sex as fixed factors, and pig and pen as random factors; interaction between diet and infection was also included in the model. For analysis of ELISA data, time was included as an additional fixed factor to account for repeated measurements. Non-normally distributed data was analyzed with a general linear model, which included diet, infection and sex as fixed factors.

## Results

### Effect of dietary inulin on peripheral immune responses during *Trichuris suis* infection

**Figure 1:**
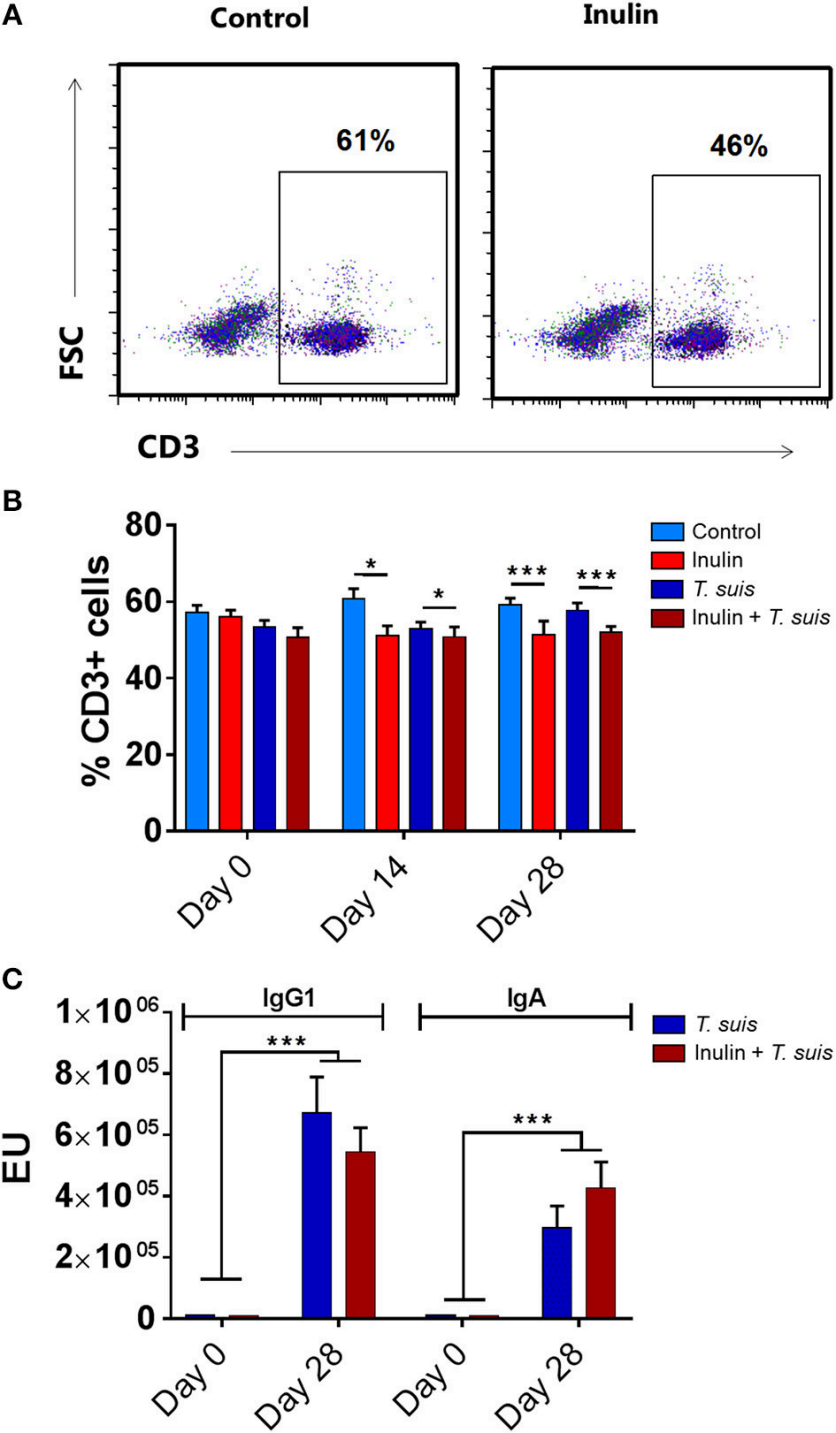
Diet supplementation with inulin alters key immune parameters. Flow cytometric analysis was conducted on peripheral blood mononuclear cells (PBMCs) isolated from heparinised blood samples attained from all animals (*n* = 31) prior to *Trichuris suis* infection (day 0), and days 14 and 28 post-infection (p.i.). **(A)** Representative flow cytometry plots of PBMC-derived CD3^+^ T cells from control-fed and inulin-fed pigs. Numbers represent the percentage of CD3^+^ T cells. **(B)** Percentage CD3^+^ T cells derived from total PBMC population. **(C)** Serum antibody levels against *T. suis* excretory/secretory antigen, expressed as ELISA units (EU). Antibody levels were measured weekly from day 0 to 28 p.i. Data are presented as means and error bars represent SEM (*p ≤ 0.05, ***p ≤ 0.005, by mixed linear model).

We first assessed whether dietary inulin modulated peripheral indicators of immune function following inoculation with *T. suis*. Independently of infection, the leukocyte profile in PBMCs showed that dietary inulin significantly decreased the proportion of CD3^+^ T cells at day 14 (*p* < 0.05) and day 28 p.i. (*p* < 0.005, Figures 1A,B). Neither diet nor infection significantly affected proportions of CD3^+^CD4^+^ helper T cells, CD3^+^CD8^+^ cytotoxic T cell populations. Similarly, no significant influence of diet or infection was observed on peripheral monocyte and B-cell populations (Figure S3).

Serum antibody levels were measured weekly from day 0 to 28 p.i., with all pigs sera negative at day 0. As expected, infection with *T. suis* significantly raised *T. suis-*specific IgA (day 21 p.i., *p* < 0.005; and day 28 p.i., *p* < 0.005) and IgG_1_ (day 28 p.i., *p* < 0.005) antibody titer levels compared to uninfected pigs. No significant effect of diet was observed on *T. suis*-specific IgA or IgG_1_ levels at any time point during the study (Figure 1C).

Thus, dietary inulin appears to modulate peripheral T-cell populations independent of infection status, whereas proportions of peripheral B cells, monocytes and acquisition of parasite-specific antibodies were not significantly affected.

### *Trichuris suis* infection induces localized type-2 immune responses and morphological changes

After 28 days of infection, pigs were necropsied and successful *T. suis* colonization was confirmed with larval burdens of 4,352 ± 2,079 (mean ± SD) and 3,838 ± 1,020 (mean ± SD) in control-fed and inulin-fed groups (*p* > 0.05). No larvae were recovered from control animals.

**Figure 2:**
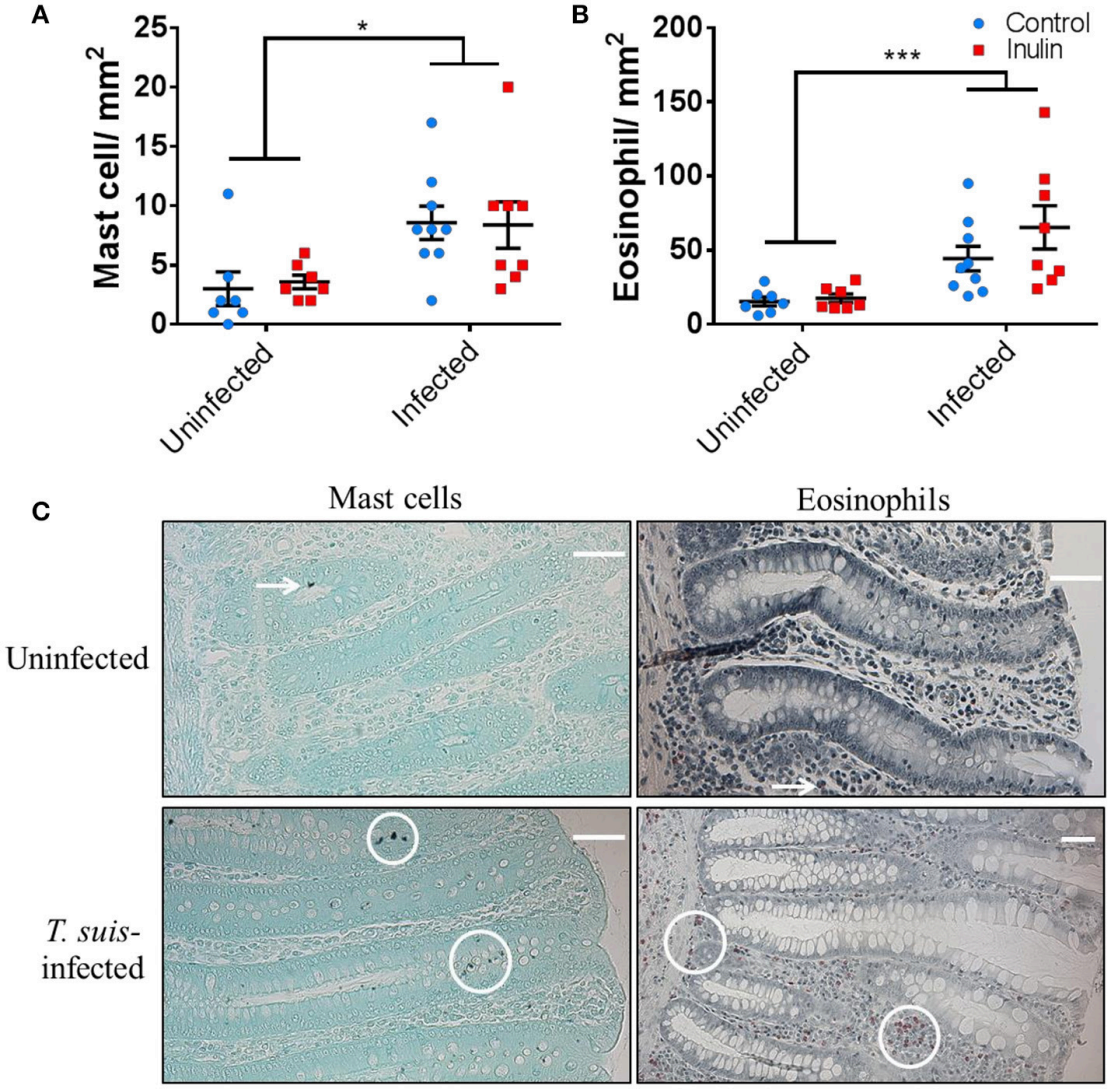
*Trichuris suis* infection modulates local gut immunology and induces histomorphological changes. Numbers of mast cells **(A)** and eosinophils **(B)** present in proximal colon tissue at day 28 post-infection. Representative histology sections show cell types visualized with Toluidine Blue and Luna stain, respectively **(C)**. Scale bars represent 50 μM. Data are presented as means and error bars represent SEM (*p ≤ 0.05, ***p ≤ 0.005, by mixed linear model).

To confirm the induction of stereotypical helminth-induced responses we assessed the modulation of local immune cells and intestinal morphology. *T. suis*-infected pigs had significant infiltration of mast cells in the proximal colon mucosa (*p* < 0.05), and tissue eosinophilia (*p* < 0.005), compared to uninfected controls (Figures 2A–C); there was no effect of diet on mast cell or eosinophil numbers. Analysis of ileo-caecal lymph node cell populations (CD3^+^, CD4^+^, CD8^+^, CD14^+^, CD79^+^) showed no significant differences between diet or infection treatments (Figures S4A–C). Stimulation of lymph node cells with *T. suis* E/S antigen elicited no IFN-γ or IL-10 cytokine secretion (*p* > 0.05), whereas mitogenic stimulation with PHA tended to increase secretion of both cytokines in the inulin-treated pigs compared to controls, although no significant differences were observed (Figures S4D,E).

### Inulin and *Trichuris suis* infection modulate the prokaryotic gut microbiota

The prokaryotic gut microbiota composition is closely linked to and has a dynamic relationship with mucosal immune function (29). Since *T. suis* infection induced structural and immunological changes in the proximal colon, we next investigated changes in the microbiota composition in this location, induced by *T. suis* and/or inulin.

**Figure 3:**
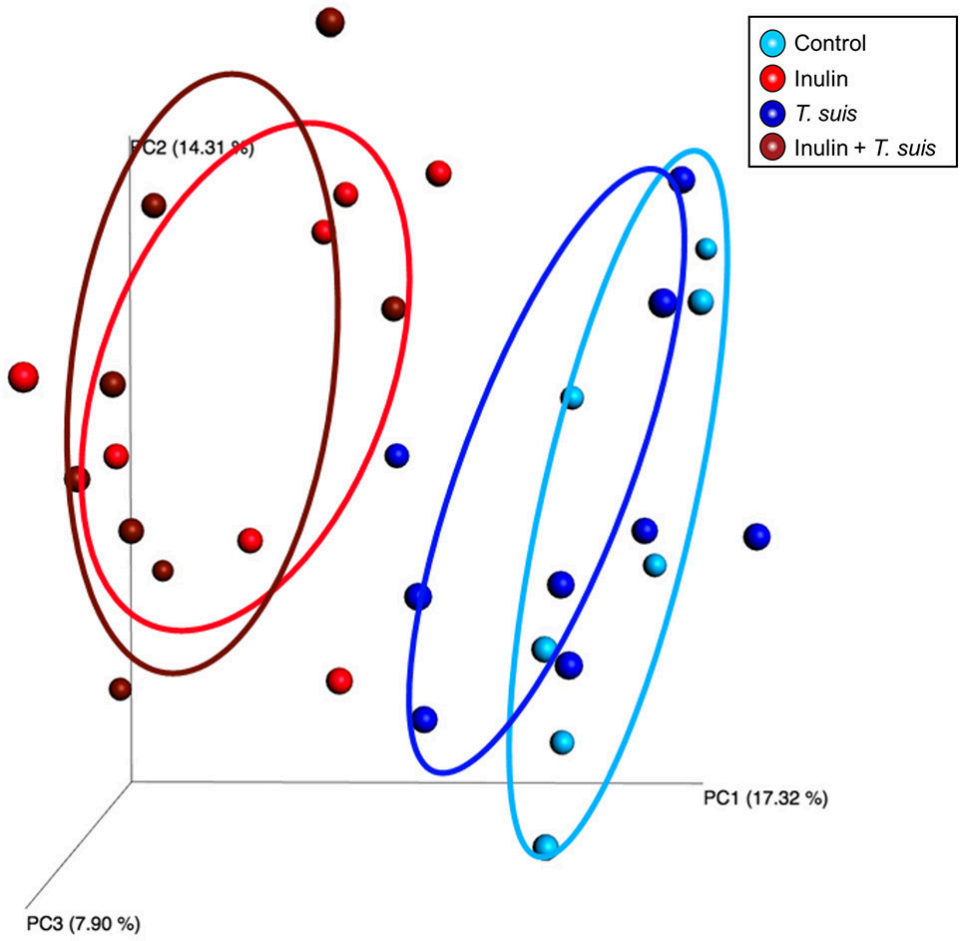
Beta diversity of bacterial taxa in proximal colon. Principal coordinates analysis (Sorensen-Dice distance matrix) of digesta samples from the four treatment groups: Control (*n* = 7); Inulin (*n* = 7); *T. suis* (*n* = 9); Inulin + *T. suis* (*n* = 8).

Alpha diversity showed no difference in the number of species in the four treatment groups (data not shown). The Beta diversity based on presence and absence of bacterial species (Sorensen-Dice distance matrix), and abundance of species (Bray-Curtis distance matrix), showed a clear separation between inulin-fed and control-fed groups (ANOSIM, Sorensen-Dice, *p* < 0.05, Figure 3). The effect of *T. suis* infection was less clear, with no significant difference for abundance of species (ANOSIM, Bray-Curtis, *p* > 0.05). There was however a significant difference in presence/absence of species between infected and uninfected pigs within the control diet (ANOSIM, Sorensen-Dice, *p* < 0.05); indicating the changes in the microbial composition following *T. suis* infection mainly affected the low-abundance species. The effect of infection was less pronounced in the inulin-fed groups (*p* > 0.05), however the shift in microbiota composition induced by *T. suis* tended to resemble that induced by inulin, such that the group most divergent from the control group were those pigs fed inulin and infected with *T. suis* (Figure 3).

**Figure 4:**
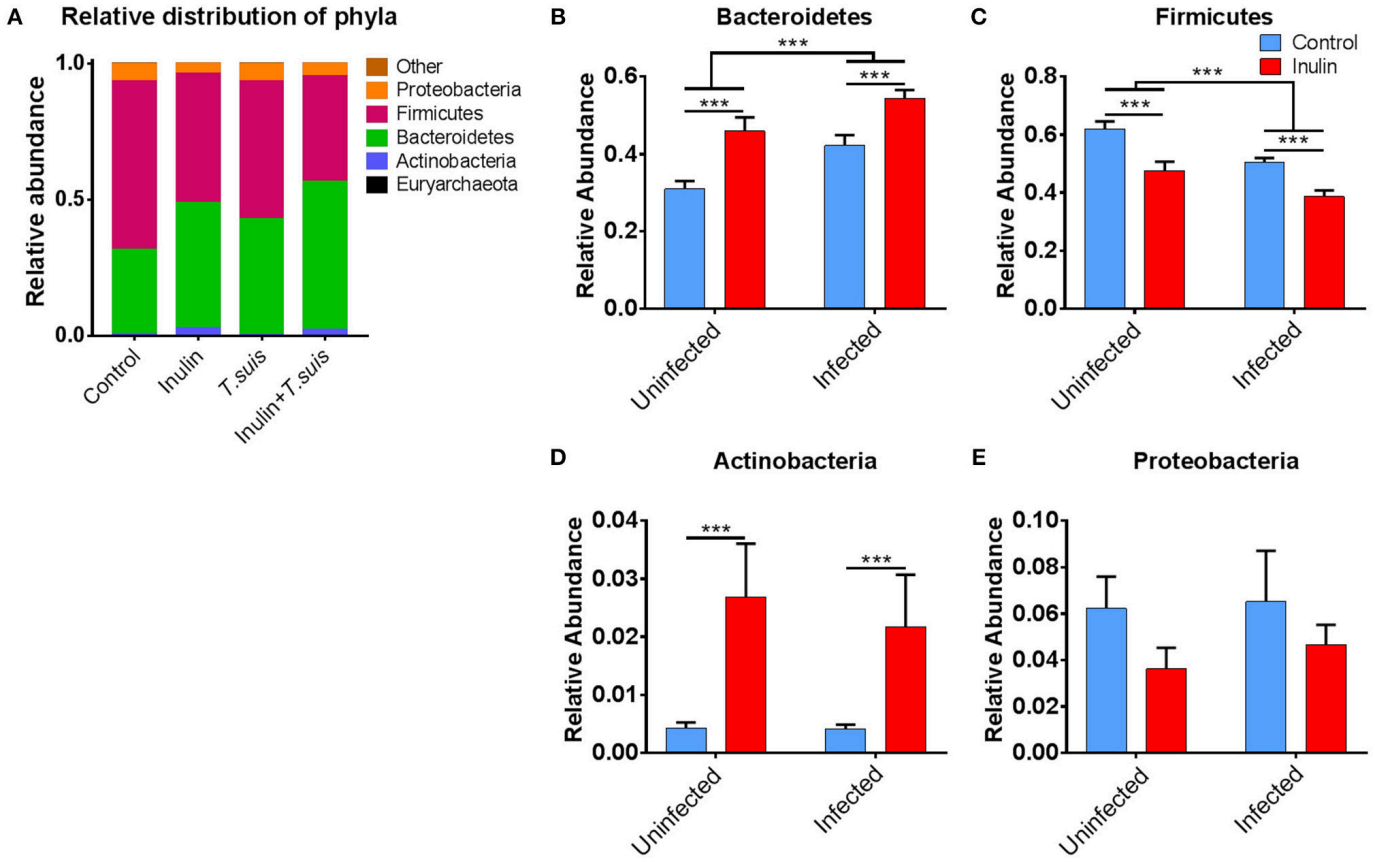
Significant alterations in microbiota at phylum level. Relative distribution of phyla in the proximal colon at day 28 post-infection **(A)**, and alterations induced by diet and *Trichuris suis* infection in **(B)** Bacteroidetes; **(C)** Firmicutes; **(D)** Actinobacteria; and **(E)** Proteobacteria phyla. Data are presented as means and error bars represent SEM (***p ≤ 0.005, by mixed linear model).

As shown in Figure 4A, closer inspection of the bacterial taxa modified by diet and/or *T. suis* revealed that the Bacteroidetes phylum increased in both inulin-fed pigs, 30.9 to 46.0% (*p* < 0.005), and those infected with *T. suis*, 30.9 to 42.2% (*p* < 0.005), compared to controls (Figure 4B). Conversely, the proportion of Firmicutes decreased in relative abundance from 62.1 to 47.6% (*p* < 0.005) for inulin-fed pigs, and from 62.1 to 50.6% (*p* < 0.005) in the *T. suis-*infected pigs (Figure 4C). Thus, the highest Bacteroidetes: Firmicutes ratio was in those pigs fed inulin and infected with *T. suis*, suggesting that both diet and *T. suis* may alter the profile of gut microbes linked with the onset of inflammation, as a low Bacteroidetes: Firmicutes ratio is considered to be associated with inflammatory diseases and obesity (30). Dietary inulin also increased the proportion of the Actinobacteria phylum from 0.4 to 2.7% (*p* < 0.005, Figure 4D), and decreased the proportion of the Proteobacteria phylum from 6.2 to 3.6% (*p* = 0.088, Figure 4E). No significant interactions between diet and infection were observed on microbiota at the phylum level.

The changes observed in relative abundance of the Bacteroidetes, Actinobacteria and Proteobacteria phyla could be assigned to genus level (Figure S5). The rise in Bacteroidetes was derived almost exclusively from an increase in *Prevotella*, a genus often associated with a very fiber-rich diet (31). Inulin supplementation increased the proportion of *Prevotella* from 8.9 to 16.8%, compared to the control-fed group, although this difference was not statistically significant. Similarly, the change in Actinobacteria could also be attributed to a single genus—*Bifidobacterium*. A higher abundance of *Bifidobacterium* is often associated with inulin supplementation (32), as observed in the inulin-fed group (2.4%) compared to controls (0.2%, *p* < 0.01). The decrease in Proteobacteria was driven by a significant reduction in *Campylobacter*, from 2.8% in the control group, to 0.1% in the inulin-fed group (*p* < 0.005). The changes within the Firmicutes phylum were more diverse (Figure S6), with dietary inulin reducing the abundance of several families, primarily *Lachnospiraceae* (*p* < 0.005)*, Clostridiaceae* (*p* < 0.05)*, Ruminococcaceae* (*p* < 0.05), whilst increasing the *Erysipelotrichaceae* (*p* < 0.005), and *Veillonellaceae* (*p* = 0.068). The rise in *Erysipelotrichaceae* was mostly associated with an increase in the genus *Catenibacterium*, which previously has been reported to associate with inulin-supplementation (33), whereas the increase in *Veillonellaceae* derives mostly from an increase in *Dialister, Megasphara* and *Mitsuokella*. The most notable difference in the *T. suis*-infected pigs was an increase in *Lactobacillaceae* from 5.1 to 8.0%, albeit this was not statistically significant. This increase was mostly due to an increase in the species *L. crispatus* from 0.7 to 2.0% and *L. kitasatonis* from 0.5 to 1.3%; the remaining species were unclassified.

**Figure 5:**
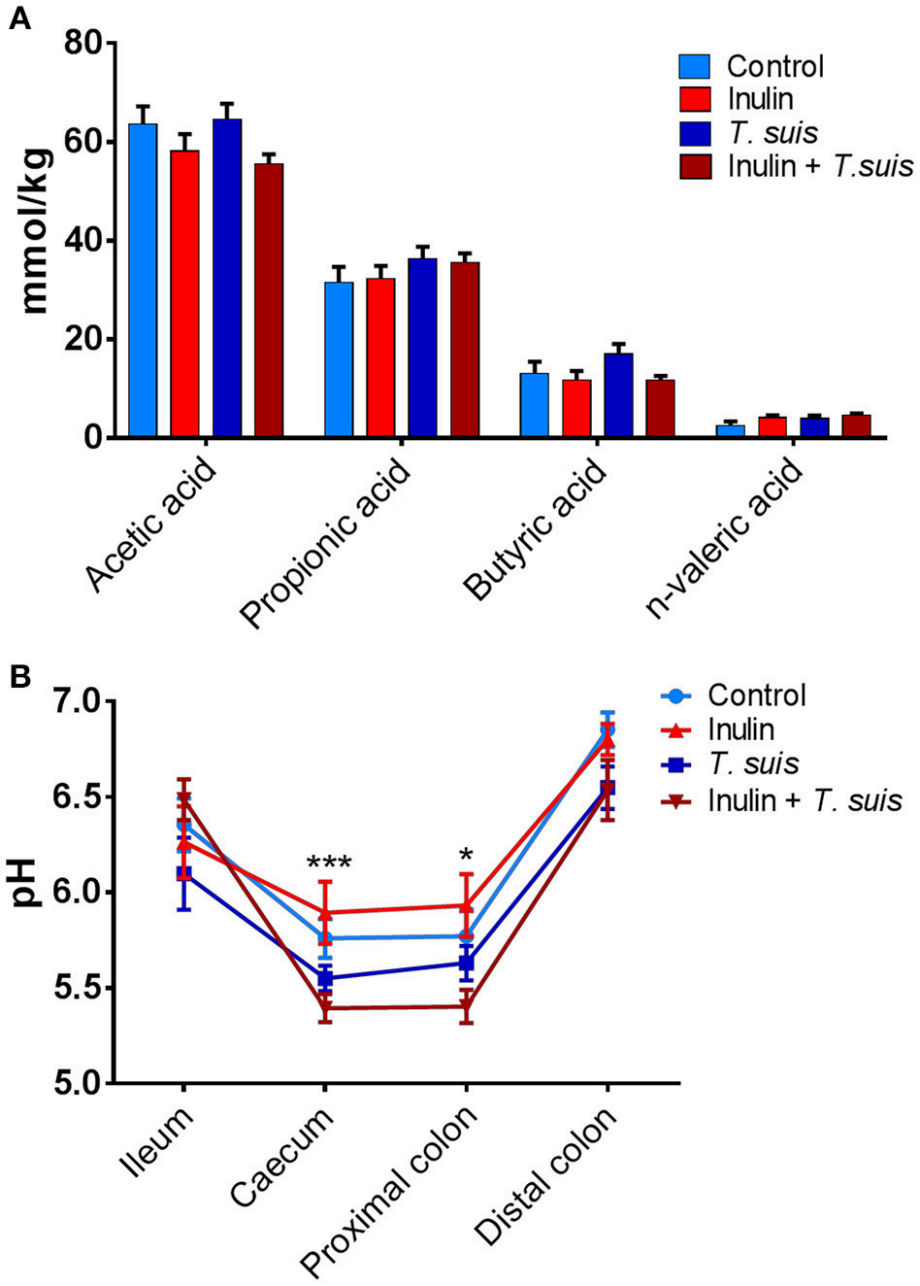
Diet and *Trichuris suis*-mediated effects on gut physiology. **(A)** Short-chain fatty acid (SCFA) profiles from proximal colon digesta samples were quantified by gas chromatography. **(B)** pH measurements from digesta collected from four intestinal locations: ileum, caecum, proximal and distal colon, with significant effect of infection observed at both caecum and proximal colon. Data are presented as means and error bars represent SEM (*p ≤ 0.05, ***p ≤ 0.005, by mixed model).

To investigate possible functional consequences of the changes in microbiota composition, we measured the production of SCFAs and lactic acid in the proximal colon, as well as pH throughout the ileum and large intestine. The abundance of acetic acid (*p* = 0.087) and n-valeric acid (*p* = 0.081) tended to be affected by diet, with acetic acid abundance being lower in inulin-fed pigs and n-valeric acid being higher in inulin-fed pigs, compared to control-fed groups (Figure 5A). However, neither diet nor infection significantly affected the levels of any of the measured SCFA (such as propionic or butyric acid). Dietary inulin also did not affect pH concentrations in the gut. In contrast, infection had a significant effect on caecal (*p* < 0.005) and proximal colon pH (*p* < 0.05), with the inulin + *T. suis* treatment group having the lowest pH in both the caecum and proximal colon, compared to other treatment groups (Figure 5B).

### Dietary inulin and *Trichuris suis* infection synergistically boost expression of th2- and mucosal function-related immune genes

**Figure 6:**
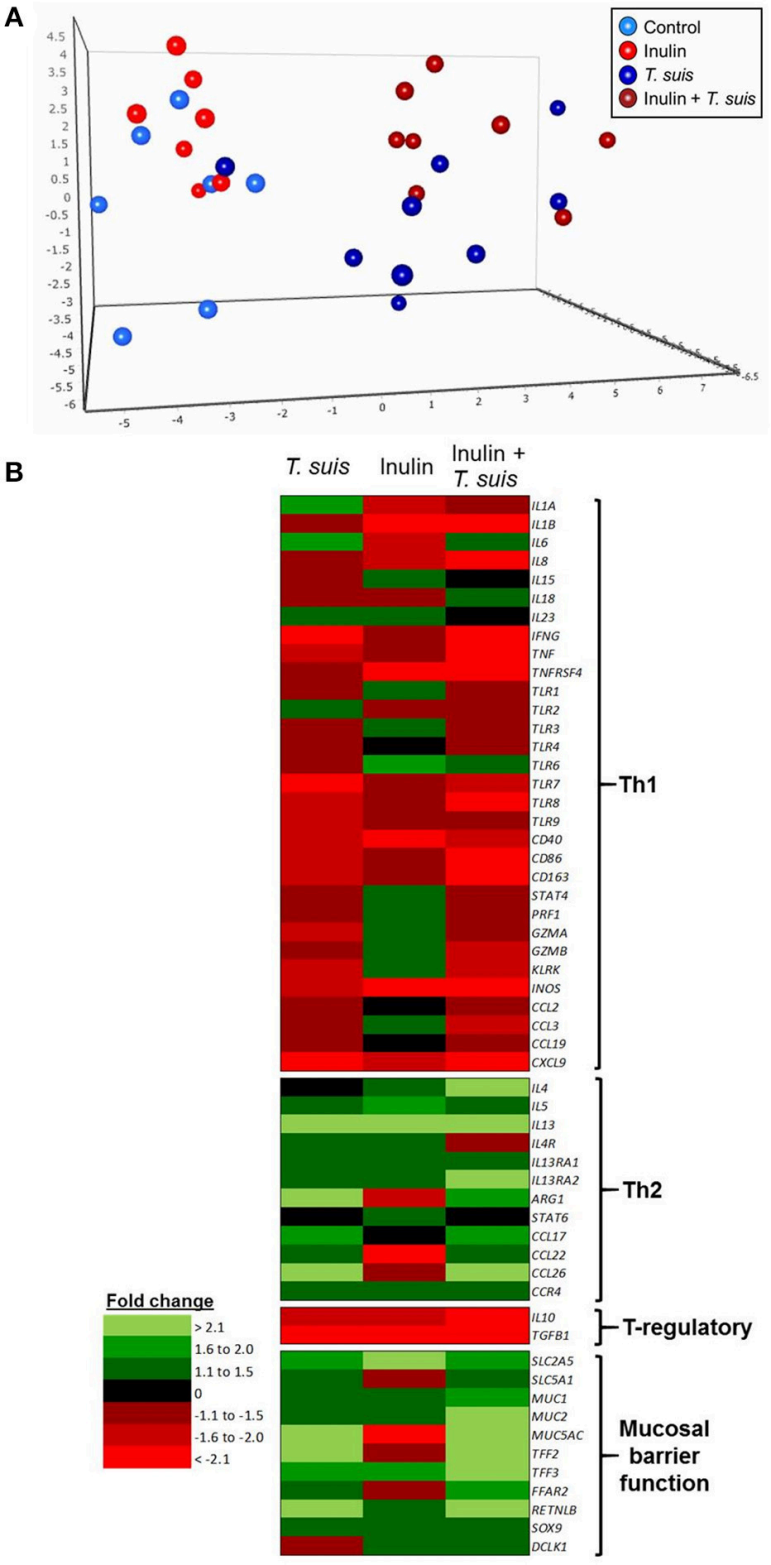
Diet and helminth infection greatly influences intestinal gene expression profiles. QPCR gene expression analysis of proximal colon tissue obtained at day 28 post-infection: **(A)** Principal component analysis of the relative expression of all immune genes (excluding housekeeping genes) from the four treatment groups: Control (*n* = 7); Inulin (*n* = 7); *T. suis* (*n* = 9); Inulin + *T. suis* (*n* = 8). **(B)** Fold change heat map of all immune genes analyzed. All fold change data are relative to the uninfected, control-fed group.

Interactions between diet and the gut microbiota likely have a pronounced effect on mucosal immune function, and so we investigated in detail how dietary inulin and/or *T. suis* infection modified the transcription of immune, inflammation and mucosal barrier-related genes in the proximal colon. Overall, diet and infection both induced distinct immune phenotypes, and once again the *T. suis*-infected pigs fed inulin were the most divergent from control pigs, as evidenced by principal component analysis (PCA; Figure 6A). Inspection of the transcriptional profile indicated that both dietary inulin and *T. suis* down-regulated Th1-related genes and up-regulated Th2- and mucosal barrier response-related genes, indicating a remarkably similar modulatory effect (Figure 6B). Treatment with either inulin or *T. suis* infection induced significant changes in the expression of 18–25 key immune genes, respectively (Table S3).

**Figure 7:**
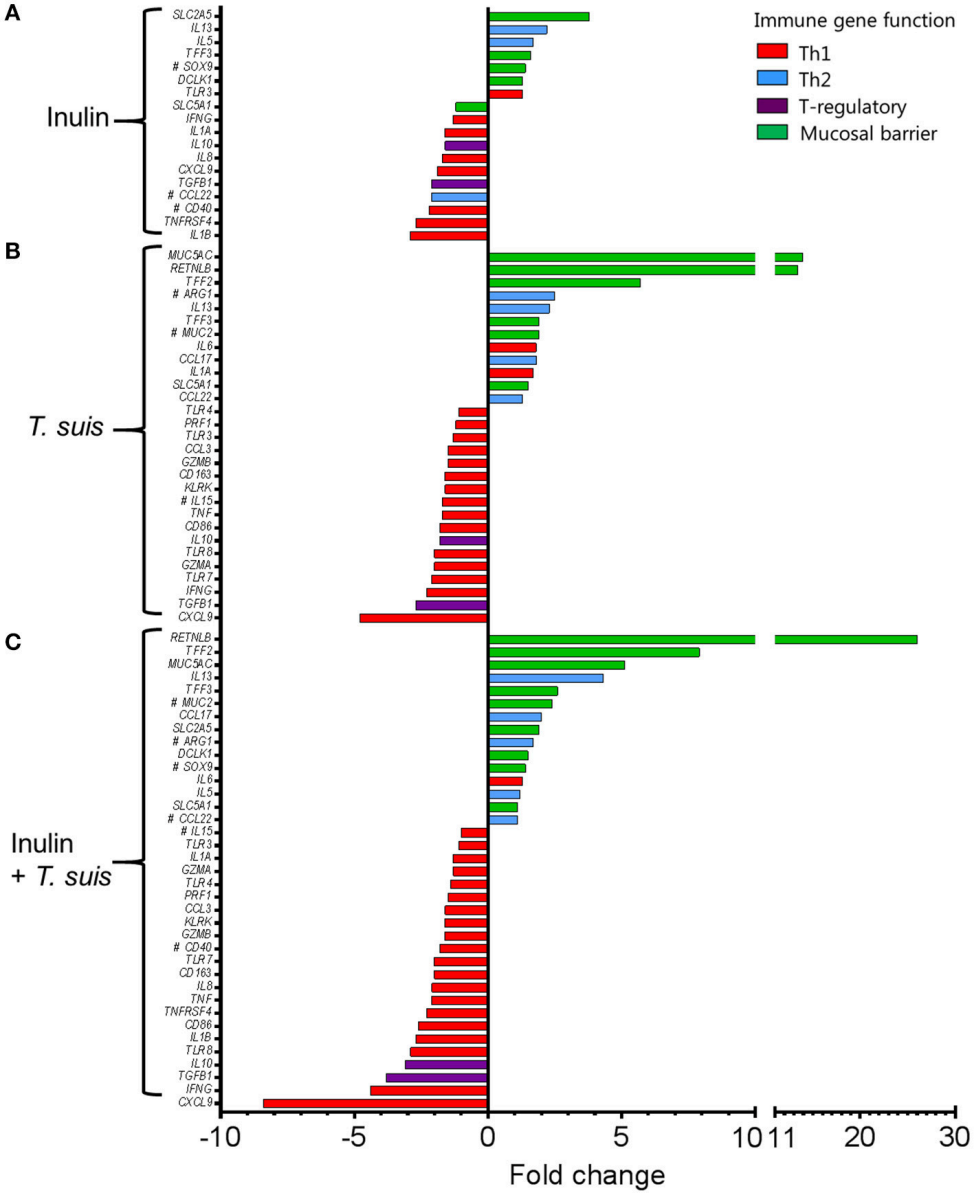
Intestinal gene expression is significantly altered by diet and/or helminth infection. Shown is the expression of all statistically significant genes (*p* ≤ 0.05) compared to control pigs fed a basal diet and without *Trichuris suis* infection. **(A)** Expression of diet-influenced genes in inulin-fed pigs without infection. **(B)** Expression of infection-influenced genes in *T. suis-*infected pigs fed a basal diet. **(C)** Expression of diet or infection-influenced genes in pigs fed inulin and infected with *T. suis*. ^#^*p* < 0.1, indicates a trend toward statistical significance, determined by mixed model.

Dietary inulin supplementation resulted in activation of genes encoding the Th2 cytokines *IL13* (*p* < 0.005) and *IL5* (*p* < 0.05). Additional mucosal responses were also activated as indicated by up-regulation of *TFF3* (*p* < 0.005), the epithelial transporter gene *SLC2A5* (*p* < 0.01), and the epithelial tuft cell markers *DCLK1* (*p* < 0.005) and *SOX9* (*p* = 0.074). Similarly to the *T. suis* only treated group, inulin–fed pigs displayed suppression of classic Th1 and regulatory immune genes, such as *IFNG* (*p* < 0.05), *IL8* (*p* < 0.01), *IL10* (*p* < 0.05), and *TGFB1*(*p* < 0.05, Figure 7A).

The *T. suis*-infected pigs displayed a characteristic polarized Th2 immune response (Figure 7B), with typical down-regulation of key Th1 immune-related genes, such as *IFNG* (*p* < 0.005) and the pro-inflammatory chemokine *CXCL9* (*p* < 0.005), as well as T-regulatory-related genes *IL10* (*p* < 0.005) and *TGFB1* (*p* < 0.05). Infection with *T. suis* also increased mucosal barrier function-related gene expression with fold changes > 12 for *RETNLB* (*p* < 0.005), compared to the control group, and up-regulation of other helminth infection-related genes, such as the mucin *MUC5AC* (*p* < 0.01), Th2 cytokine *IL13* (*p* < 0.005), and goblet cell trefoil factors *TFF2* (*p* < 0.01), *TFF3* (*p* < 0.005), indicating activation of local Th2 mucosal responses.

**Figure 8:**
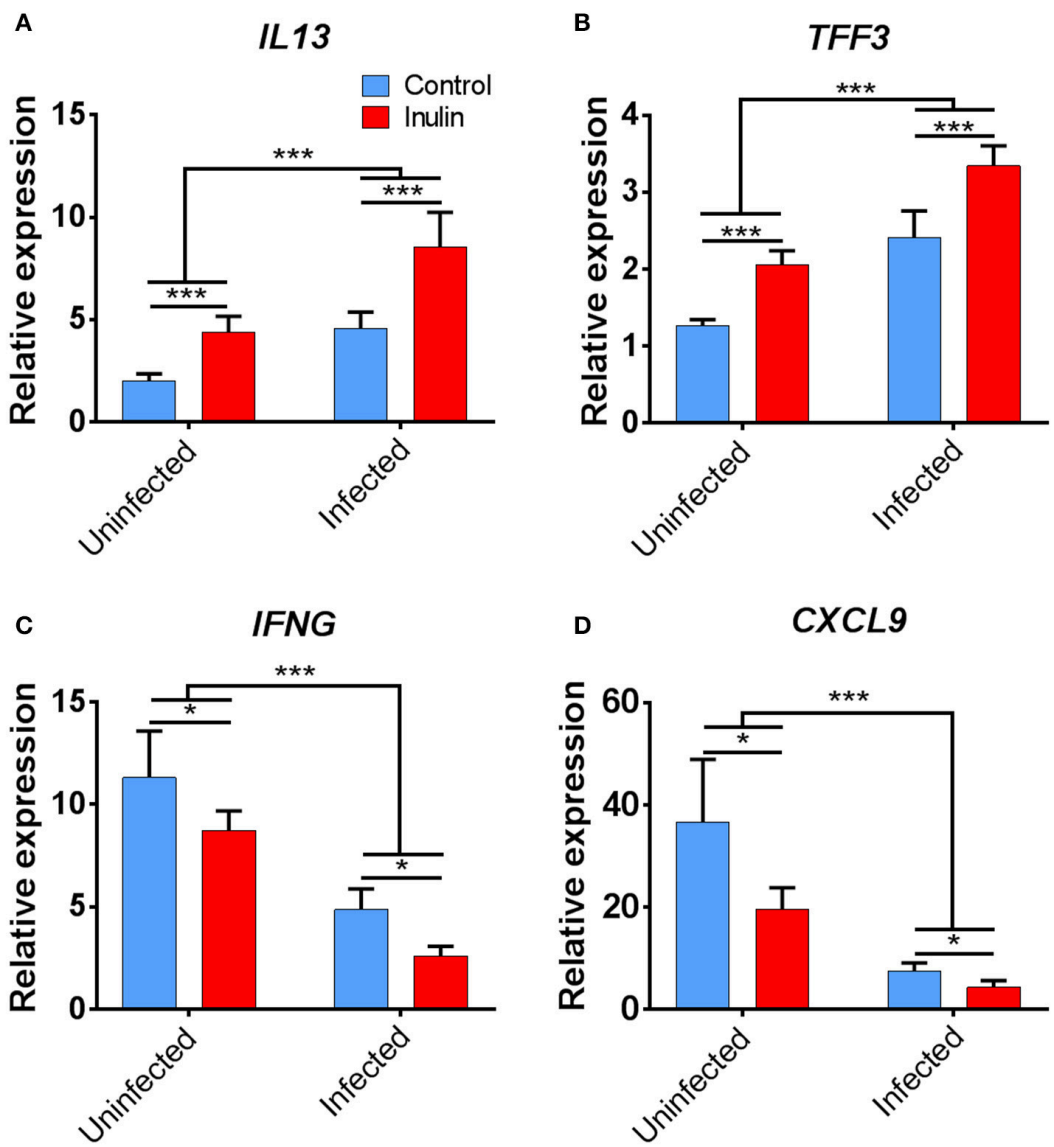
Key immune genes in colon tissue differentially affected by treatment. Relevant expression of key immune genes: **(A)** *IL13*; **(B)** *TFF3*; **(C)** *IFNG*; and **(D)** *CXCL9*. Data are presented as means and error bars represent SEM (*p ≤ 0.05, ***p ≤ 0.005, by mixed model).

Treatment with both dietary inulin and *T. suis* infection appeared to boost expression of both Th2 and mucosal barrier-related genes, compared to either treatment in isolation (Figures 7C). For example, *T. suis* induced-changes in expression of *IL13* (fold change>4), and *TFF3* (fold change >2) were all increased by concurrent inulin intake, as illustrated in Figures 8A,B, respectively. In addition, expression of the epithelial tuft cell markers *DCLK1* and *SOX9* were increased in *T. suis*-infected pigs fed dietary inulin, highlighting the possible role of inulin in Th2 polarization, as tuft cells have recently been shown to be crucial players in initiation of Th2 responses during helminth infection in mice (34). Finally, down-regulation of pro-inflammatory genes, such as *IFNG* (fold change <−4, Figure 8C) and *CXCL9* (fold change <−8, Figure 8D) in *T. suis-*infected pigs was further decreased by inulin. Overall, the transcriptional data from the colon indicates that both *T. suis* and inulin suppress expression of pro-inflammatory and Th1 related genes whilst enhancing expression of Th2 and mucosal barrier genes, indicating that dietary inulin augmented the Th2 polarized environment during helminth colonization.

### Effects of dietary inulin and *Trichuris suis* infection on goblet and tuft cell abundance in the proximal colon

**Figure 9:**
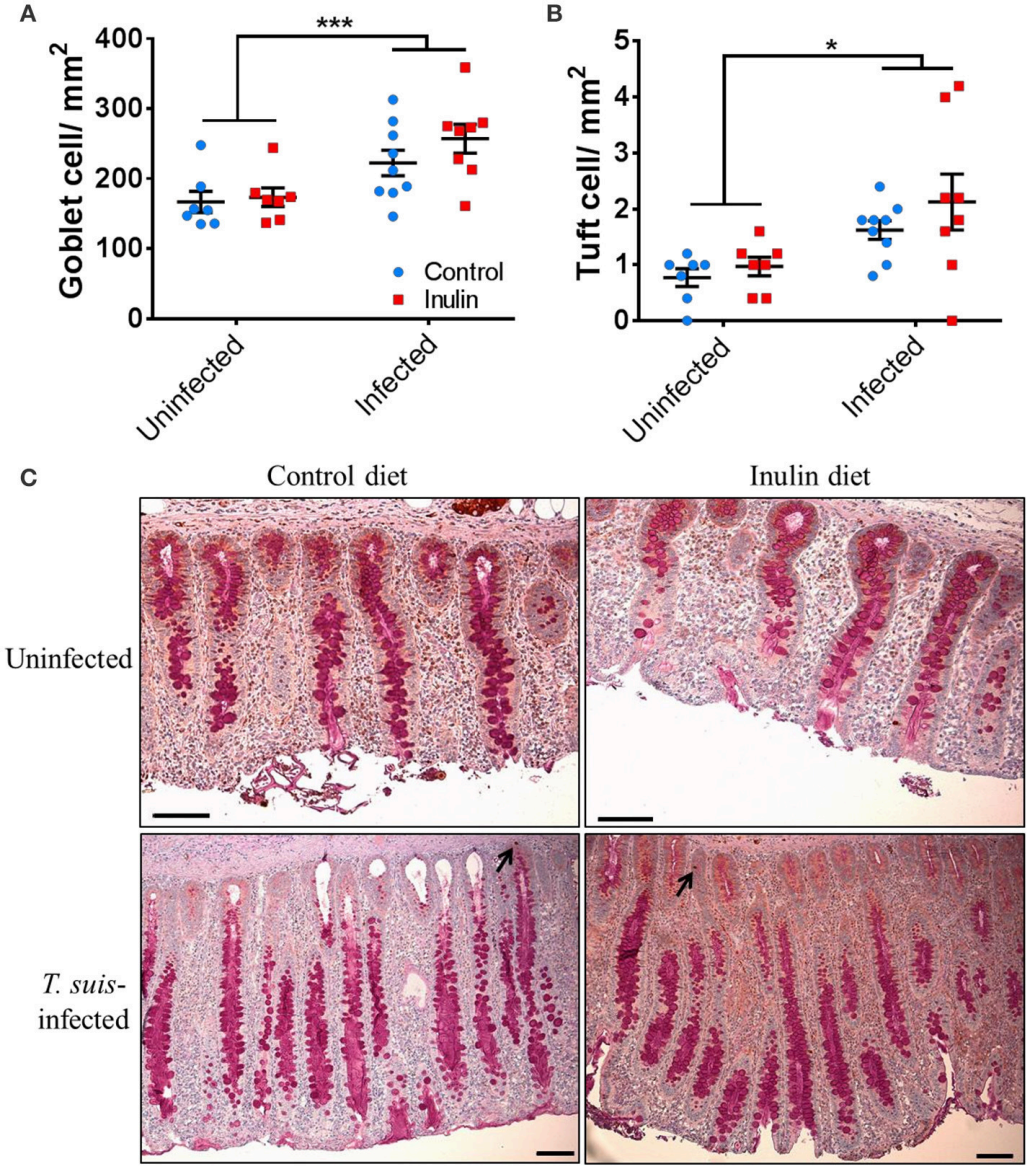
Morphological changes to the mucosal barrier associated with *Trichuris suis* infection. Representative histological staining for mucins present in goblet cells **(A,C)**, and immunohistochemical staining for quantification of tuft cells present in the epithelial crypts, as indicated by arrows **(B,C)**. Scale bars represent 50 μM. Data are presented as means and error bars represent SEM (*p ≤ 0.05, ***p ≤ 0.005, by mixed linear model).

The transcriptional changes within the proximal colon which indicated an up-regulation of goblet and tuft cell-related markers led us to investigate changes in these cell numbers. Goblet cell numbers were higher in *T. suis*-infected pigs compared to uninfected pigs (*p* < 0.005, Figures 9A,C). In addition, epithelial tuft cell number was significantly increased in infected pigs, compared to uninfected controls (*p* < 0.05, Figures 9B,C). Numbers of both cell types were slightly higher in infected pigs supplemented with inulin; however the effect of diet was not statistically significant. In addition, morphological changes associated with *T. suis* infection were also seen, such as elongation of epithelial crypts and hypertrophy of the mucosa in the proximal colon (Figure 9C).

## Discussion

The interaction between dietary components and intestinal microbes on mucosal immune function is becoming increasingly relevant due to rising rates of chronic inflammatory disorders. In the present study, we observed that both dietary inulin and helminth infection generally activated host Th2 immune responses, and in turn suppressed Th1 inflammatory responses. Moreover, concurrent inulin supplementation and *T. suis* infection appeared to co-operatively enhance expression of key mucosal barrier function-related genes, indicating a mechanism whereby diet may augment specific mucosal immune function and gut health. Furthermore, consistent with previous studies, both experimental helminth infection and inulin treatment altered commensal microbial composition in the gut.

The up-regulation of Th2 genes in the gut indicated the typical Th2 immune activation that is associated with helminth infection, therefore supporting the relevance of the *T. suis*-pig model for investigating dietary immunomodulation of naturally-induced Th2 responses. As expected, due to the invasive nature of the establishing helminth infection in the tissue mucosa, a clear effect of infection was present on gut morphology, immune cells and goblet cell mucin production, which enhances the mucus layer and thus can protect the mucosal barrier from colonization by pathogenic organisms (35). In *Trichuris* infections, thickening of the mucus layer is accompanied with increased epithelial cell turnover eventually dislodging the worms from the epithelial lining and expelling them from the host (36). Helminth-induced Th2 responses not only resulted in goblet cell proliferation, but also general intestinal remodeling. Other cell types present in the mucosa were also influenced by helminth infection: for example tuft cells, a newly characterized epithelial cell type known to be stimulated by the Th2 cytokines IL-4/IL-13 in mice (37) were significantly increased in infected pigs compared to uninfected controls, thus confirming tuft cells are also induced by helminth infection in pigs. Previous studies have suggested that tuft cells are responsible for initiating helminth-specific Th2 immune responses by secreting the alarmin IL-25 (34). It is clear from the alterations in proximal colon *MUC2* gene expression that inulin is also capable of modulating the colonic mucosal environment, and may augment the *T. suis*-induced changes as evidenced by increased *RETNLB* and *TFF3* expression in infected pigs fed inulin. Whether these inulin-induced changes may eventually contribute to increased expulsion or reduced fecundity of *Trichuris* is unclear; in our study *T. suis* larval burdens were similar between control and inulin-fed pigs at day 28 p.i., and previous studies have shown conflicting results of the effect of dietary inulin on adult *T. suis* worm burdens (38, 39). Nevertheless, our results show that acquisition of type-2 immune responses were significantly enhanced by inulin treatment.

In addition to increased induction of Th2 and mucosal barrier immune responses, an equally important finding in our study was the opposing suppression of inflammatory Th1 immune responses that was clearly observed with *T. suis* infection and dietary inulin treatment. Reduction of inflammatory responses, particularly in the gut, is highly relevant for the treatment of several immune mediated diseases, such as inflammatory bowel disease. The application of helminths or helminth-derived products has shown promise in treating several autoimmune and inflammatory diseases; however conflicting reports have been obtained suggesting that refinement of this approach is needed (40). Our study indicates that inulin supplementation may have similar anti-inflammatory properties and may synergize with helminth infection, thus combination therapy with helminth products and prebiotic inulin may be a novel approach for treatment of autoimmune disorders.

Investigation of the intestinal microbiome indicated a change in microbial composition, both as a result of dietary changes and infection with *T. suis*, toward more beneficial and SCFA producing genera and fewer pathogenic species. Intake of a high fiber diet has previously been shown to increase the proportion of Bacteroidetes that release SCFA due to fiber fermentation. This fermentation subsequently lowers the pH of the gut and further impacts on microbiota composition (41). We observed minor changes in SCFA concentration between treatment groups, with a tendency of inulin to decrease acetic acid. The absence of significant changes in SCFA profiles may be a result of rapid absorption of SCFAs by host colonocytes and therefore altered levels may be undetectable despite changes in the microbiota composition. The shift in the intestinal microbiota induced by inulin was substantial, and will consequently alter both metabolism and likely the local mucosal immune responses. Thus, it is also plausible that the observed immunomodulation occurred via a microbiota-dependent pathway that is not related to fermentation by-products (SCFAs) or the resulting physico-chemical changes. The most notable and significant changes to the microbiota composition induced by inulin in the proximal colon was a 2-fold increase in *Bifidobacterium* abundance, and the relative absence of *Campylobacter* in inulin-fed pigs compared to controls. Bifidobacteria are known to modify the gut environment making it less favorable for the growth of pathogenic bacterial species (42). Negative interactions between Bifidobacteria and *Campylobacter* have been noted previously (39), as well as a reduction in *Brachyspira hyodysenteriae*-related swine dysentery thought to be mediated by an increase in Bifidobacteria in pigs fed inulin-rich chicory root (43). This may be consistent with our results suggesting that fermentation of inulin in the caecum/proximal colon enhances Bifidobacteria which outcompete pathogenic bacterial species. As a result, this may reduce Th1-stimulating pathogens, which may subsequently promote host protective Th2 responses. Alternatively, inulin may interact directly with the mucosal barrier and associated immune cells. Whilst the documented immunomodulatory effects of inulin are normally ascribed to its prebiotic effects, recent studies have suggested that inulin fibers may directly activate host immune cells in a receptor-dependent fashion (14, 44). Clearly, further studies will be necessary to unravel the role of the gut microbiota in the health-promoting effects of inulin. Notably, the shift in microbiota composition induced by *T. suis*, whilst qualitatively similar to that induced by inulin, was lower in magnitude which may suggest that the strong Th2-response induced by *T. suis* was related primarily to the direct interactions of parasite antigens with immune cells in the mucosa.

Pigs fed inulin and infected with *T. suis* had the highest abundance of Bacteroidetes to Firmicutes, which is often indicative of a more beneficial gut microbiota composition (45). Our results are consistent with others demonstrating the dynamic relationship between helminth infection and gut microbiota composition specifically in the porcine proximal colon (46, 47). The interaction between helminths and host microbiota is well-documented, with shifts in microbiota richness and composition resulting from infections with *N. americanus* (48) and *Trichuris* spp. infection (49–52). As reported by Reynolds et al. these changes to the host microbiota may even benefit the host, such as the increase in probiotic *Lactobacillaceae* observed with chronic *Heligmosomoides polygyrus* infection in mice (53); it is yet unknown whether the accompanying immunomodulation is a result of the helminth infection, or by microbiota-dependent mechanisms. However, it is known that both helminth and microbiota can elicit similar immunomodulatory effects that may have implications beyond that of localized gut immune responses. For example, Trompette et al. reported that dietary fibers can influence the severity of allergic inflammation in the lung, via activation of GPR41 by microbiota-derived SCFAs present in the gut (54). Furthermore, Zaiss et al. observed that chronic infection with the gut-dwelling nematode *H. polygyrus* protected mice against allergic asthma and inflammatory responses in the lung, via the same SCFA-dependent mechanisms (55). Inulin itself is capable of activating GPR41 and 43 (56), therefore it is plausible that the combined treatment of Th1-suppressing *T. suis* infection, and immunomodulatory prebiotic inulin, can both augment host microbiota composition and so co-operatively contribute to a healthier gut phenotype as well as an increased anti-inflammatory response in peripheral tissues, such as the lung, during allergic asthma or Th1-stimulating infection and disease. Interestingly, in this porcine model regulatory immune gene expression appeared to be down-regulated by both inulin and *T. suis*, likely as a result of the polarized Th2-immune response induced by both treatments. The production of regulatory molecules, such as IL-10 is important for maintenance of gut homeostasis, and some helminths and their ES products are known to induce regulatory responses that are key to modulating susceptibility and response to allergens (57, 58). Given the nature of the Th2-polarization in this model, further work is clearly needed to explore the implications in the context of allergic inflammation, and also in disease setting where Th1 responses are important.

In conclusion, we have demonstrated that inulin supplementation and *T. suis* infection can synergistically influence host immune responses resulting in enhanced Th2 and mucosal barrier gene expression, and suppression of inflammatory gene expression and potentially pathogenic bacterial populations. The mechanism by which this immunomodulation occurs may be a consequence of altered microbial populations, via direct manipulation of intestinal immune cells, or interplay between these two factors. Delineation of the precise mechanism(s) responsible for the observed immune modulation could reveal novel targets to treat inflammatory intestinal conditions, and expand knowledge of the interaction of diet and immune responses in pigs furthering the utility of the pig as a model for intestinal inflammatory conditions.

## Author contributions

AW, ST, HM, PN, TH, LM, and SS conceived the project and experiments. LM, SS, AW, ST, HM, PN, and TH performed the animal study. LM and SS performed all laboratory analyses. KS guided qPCR experimental design, sample processing, data analysis, and prepared Table S2. CS and LO guided 16S rRNA sequencing experimental design, sample processing and data analysis. LM, SS, and AW prepared the manuscript with input from all other authors. All authors reviewed the manuscript.

### Conflict of interest statement

The authors declare that the research was conducted in the absence of any commercial or financial relationships that could be construed as a potential conflict of interest.
