# Supplementary material for: Mucosal Barrier and Th2 Immune Responses Are Enhanced by Dietary Inulin in Pigs Infected With Trichuris suis
Source: Front Immunol. 2018 Nov 9;9:2557. doi: 10.3389/fimmu.2018.02557 (PMC6237860; doi:10.3389/fimmu.2018.02557)
Supplement: Supplementary file 1 [file Data_Sheet_1.PDF]

A

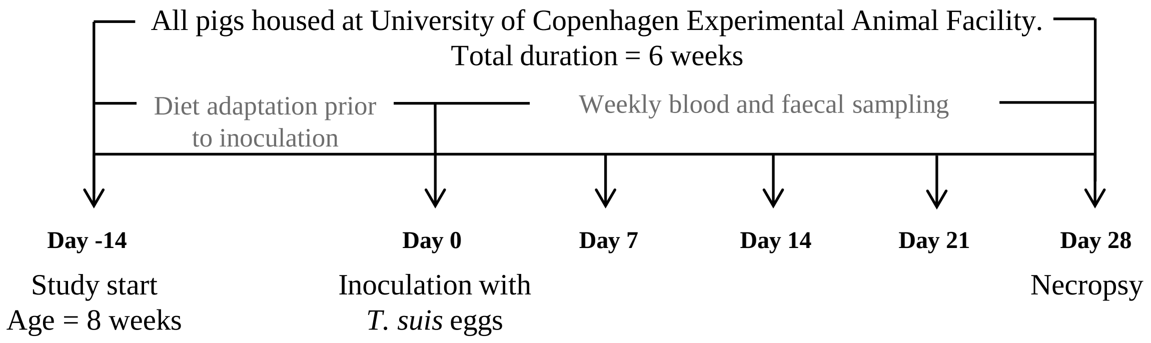

B

	**Control diet (commercial)**	**Experimental diet (10% inulin)**
**No infection**	n = 7*	n = 7*
***Trichuris suis* infection**	n = 9	n = 8*

Figure S1: (A) At study start, a total of 34 pigs (8 weeks old) were stratified according to sex and body weight, and randomly allocated into four treatment groups. All pigs were co-housed prior to allocation to experimental treatment groups. **(B)** Final number of animals per treatment group at termination of study at day 28 post-infection. * indicates reduced group number due to cause's unrelated to experimental treatment.
