# Supplementary material for: Mucosal Barrier and Th2 Immune Responses Are Enhanced by Dietary Inulin in Pigs Infected With Trichuris suis
Source: Front Immunol. 2018 Nov 9;9:2557. doi: 10.3389/fimmu.2018.02557 (PMC6237860; doi:10.3389/fimmu.2018.02557)
Supplement: Supplementary file 2 [file Data_Sheet_2.pdf]

Table S1: Composition of basal (control) and experimental inulin-supplemented diet. Both diets were pre-mixed, and balanced for crude protein and energy content. Purified long-chain inulin contained average degree of polymerization (DP) ≥ 23.

Ingredient (g/kg)	Basal (control)	Inulin
Barley	577.0	293.0
Wheat	163.0	2900
Inulin[1]	-	100.0
Soybean meal	202.0	228.0
Palm oil distillate fatty acids	15.0	30.0
Molasses	15.0	12.0
Calcium carbonate	13.0	13.0
Calcium phosphate	7.0	7.0
Sodium chloride	3.0	4.0
L-lysine hydrochloride	2.0	2.0
Methionine	1.0	1.0
Vitamin mix	2.0	2.0
**Calculated composition**		
Crude protein (g/kg)	160.0	160.0
Metabolisable energy (MJ/kg)	12.6	12.5
**Analysed composition**		
Dry matter (%)	90.7	91.1
Fat (%)	4.1	5.1
Crude fibre (%)	3.0	3.0
Ash (%)	5.2	5.1
Lysine (%)	1.0	1.0
Methionine (%)	0.3	0.3
Calcium (%)	0.7	0.7
Phosphorous (%)	0.5	0.5
Sodium (%)	0.2	0.2
Crude protein (g/kg)	162.0	173.0
Metabolisable energy (MJ/kg)	13.9	14.5

[1] Long-chain inulin purified from chicory root (Orafti®HP, Beneo, Netherlands).
