# Supplementary material for: Mucosal Barrier and Th2 Immune Responses Are Enhanced by Dietary Inulin in Pigs Infected With Trichuris suis
Source: Front Immunol. 2018 Nov 9;9:2557. doi: 10.3389/fimmu.2018.02557 (PMC6237860; doi:10.3389/fimmu.2018.02557)
Supplement: Supplementary file 3 [file Data_Sheet_3.PDF]

Table S2: qPCR gene list, primer sequences and amplicon length A panel of 91 genes of interest were analysed from proximal colon tissue RNA using Fluidigm technology, of which 56 were statistically analysed after data pre-processing.

Gene of Interest	Primer Sequence (5' to 3' forward and reverse)	Amplicon length
IL1A (Interleukin-1 alpha)	F: TGTGCTAAATAACCTGGATGAGG<br>R: GGTTCGTCTTCGTTTTGAGC	135
IL1B	F: CCAAAGAGGGACATGGAGAA<br>R: GGGCTTTTGTTCTGCTTGAG	123
IL4	F: GCAAACATGACCTGTTCTGTG<br>R: GCTTCAACACTTTGAGTATTTCTCC	105
IL5	F: GGGGAAAGATGGAGAGTAACG<br>R: CTTTCCATTGTCCACTCGGTA	83
IL6	F: TGGGTTCAATCAGGAGACCT<br>R: CAGCCTCGACATTTCCCTTA	116
IL8	F: GAAGAGAACTGAGAAGCAACAACA<br>R: TTGTGTTGGCATCTTTACTGAGA	99
IL10	F: TACAACAGGGGCTTGCTCTT<br>R: GCCAGGAAGATCAGGCAATA	110
IL13	F: CCAAGCGAGCAAGTTCCTG<br>R: AACTACCCGTGGCGAAAAAT	110
IL15	F: CGTCATTTTGCAAGAGTCCA<br>R: TGGACGATAAACTGCTGTTTGC	86
IL18	F: CAATTGCATCAGCTTTGTGG<br>R: TCCAGGTCCTCATCGTTTTC	78
IL23	F: GCTGTGATCCTCAGGGACTC<br>R: TAGAGAAGGCTCCCCTGTGA	119
CXCL9 (Chemokine (C-X-C motif) ligand 9	F: AGCAGTGTTGCCTTGCTTTT<br>R: ATGCAGGAACAACGTCCATT	92
IFNG (Interferon-gamma)	F:CCATTCAAAGGAGCATGGAT<br>R: TTCAGTTTCCCAGAGCTACCA	76
TNF (Tumour necrosis factor-alpha)	F:CCCCCAGAAGGAAGAGTTTC<br>R: CGGGCTTATCTGAGGTTTGA	92
TGFB1 (Transforming growth factor-beta)	F:TCACCGGGGCTGTATTTAAG<br>R: AAGGAAGACCCCAGTCAGGT	110
IL4R (Interleukin-4 receptor)	F: CAGAGCTGCCTGCTGTCAT<br>R: CTCTCCGGGATCTGAGGACT	80
IL13RA1 (Interleukin-13 receptor alpha 1)	F: TCCCTCCAATTCCTGATCCT<br>R: TCCAGTGCAGGGTATCATCA	75
IL13RA2 (Interleukin-13 receptor alpha 2)	F: TGAAAGCTGGAAGACGATCA<br>R: GCCCTGGCAGAAGTGTATGT	101
TNFRSF4 (TNF receptor superfamily 4)	F: CACAGGGCCTGGAGACTG<br>R: CGTGCTCCTCTTGGATGG	82
TLR1 (Toll-like receptor 1)	F: CCTTCAAGACCTTAACACACAGAG<br>R: CAGATTTACTGCGGTGCTGA	100
TLR2	F: CGGAGGTTGCATATTCCACAG<br>R: TGTGAAAGGGAACAGGGAAC	128

Table S2 continued (2/3)

Gene of Interest	Primer Sequence (5' to 3' forward and reverse)	Amplicon length
TLR3	F: ATTGTGCAAAAGATTCAAGGTG<br>R: TCTTCGCAAACAGAGTGCAT	130
TLR4	F: TGGTGTCCCAGCACTTCATA<br>R: CAACTTCTGCAGGACGATGA	116
TLR6	F: TGGATGTTAGCTCGAATTCTTTG<br>R: GAACCTTGATCCTGGGAGGT	141
TLR7	F: AGAAGCCCCTTCAGAAGTCC<br>R: GGTGAGCCTGTGGATTTGTT	93
TLR8	F: GCAAAGACCACCACCAACTT<br>R: ATCCGTCAGTCTGGGAATTG	129
TLR9	F: CCTGTTCTATGATGCCTTCGTG<br>R: GGTACCCAGTCTCGCTCCTC	144
CD40 (Cluster of differentiation 40)	F: TGAGAGCCCTGGTGGTTATC<br>R: GCTCCTTGGTCACCTTTCTG	90
CD86	F: CATCGTCTGTGTCCTGCAAC<br>R: CACAGGTGGCTTTGCATCTA	82
CD163	F:CACATGTGCCAACAAAATAAGAC<br>R: CACCACCTGAGCATCTTCAA	130
CCL2 (Chemokine (C-C motif) ligand 2)	F:CTTCTGCACCCAGGTCCTT<br>R: CGCTGCATCGAGATCTTCTT	93
CCL3	F: CTCTGCAGCCAGGTCTTCTC<br>R: CTACGAATTTGCGAGGAAGC	97
CCL17	F: GGGTGGTACCAGACCTCAGA<br>R: GTCCTTGGGGTCAGAACAGA	90
CCL19	F: CTGGACTTCTCCTGCTCTGG<br>R: AAAGGCTCGAACCAGATTCC	97
CCL22	F: CCCTGCGTGTGGTGAAGTAT<br>R: ATCTCTCGGTCCCTCAAGGT	88
CCL26	F: CTGCTTCCAATACAGCCACA<br>R: AGCAGCTGTTCCTGGTGAAT	74
CCR4 (Chemokine receptor 4)	F: GGACCCCTTACAATGTGGTG<br>R: GAATGGCGTAGTCCAGGTGT	96
ARG1 (Arginine 1)	F: TCCAAGGTCTGTGGGAAAAG<br>R: ATCGCCATACTGTGGTCTCC	108
STAT4 (Signal transducer and activator of transcription-4)	F: CCCATCTCAACAATCCGAAG<br>R: TTGGCGACATAGGAAGAAGG	70
STAT6	F: TCCCAGATGTATCCACCACA<br>R: ATCTGCAGGTGAGGTTCCTG	107
PRF1 (Perforin 1)	F: CTATGGCTGGGACGATGACC<br>R: CATGGTTCAAGGCGCACATC	86

Table S2 continued (3/3)

Gene of Interest	Primer Sequence	Amplicon length
GZMA (Granzyme A)	F: AAGGGGATCTTCAGCTGCTT<br>R: GGGGTTCGACATCTTTTCCT	99
GZMB	F: CCAGGACCAGGATAATCGAA<br>R: GGGTGACGTTGATTGAGCTT	101
KLRK (Killer cell lectin like receptor K1)	F: GATGGTTCCATCCTCTCACC<br>R: TGAGCCATAGACTGCACAGC	75
INOS (Inducible nitric oxide synthase)	F: CAGCCCAAGGTCTATGTTCAAG<br>R: ATAGAGGTGGCCTTGCTCCT	90
SLC2A5 (Solute carrier family 2 member 5)	F: GGTCATCTCCACCATCATCC<br>R: GCGCTCAGGTAGATCTGGTC	90
SLC5A1	F: TCTCATGAGCTCCCTGACCT<br>R: CTCTCTTCCGGATCTTGGTG	83
MUC1 (Mucin 1)	F: GGATTTCTGAATTGTTTTTGCAG<br>R: ACTGTCTTGGAAGGCCAGAA	116
MUC2	F: GCACGTCTGCAACAAGGAC<br>R: CAAAGCCCTCCAGGCAGT	125
MUC5AC	F: CCCAGATCTGCAGCACCTAC<br>R: GTAACACAGGCCACCTGCTT	94
TFF2 (Trefoil factor-2)	F: GCTGCTTCGACTCCCAAGT<br>R: CATGACGCACTCCTCAGACT	80
TFF3	F: TGTTCTGGCTGCTAGTGGTG<br>R: CAGTCCACCCTGTCCTTGG	112
FFAR2 (Free fatty acid receptor-2)	F: GCTTCGGGCCCTATAACATA<br>R: GCGTTGAGGGAGCTGAATAC	97
RETNLB (Resistin-like beta)	F: TCCCTCTGCTCCAAGAAAGA<br>R: CAAGCACAGCCAGTGACAAC	99
SOX9 (Sry-related transcription factor 9)	F: GACTCGCCGCACTCCTC<br>R: GTGGTGGGTGGCGTTG	92
DCLK1 (Double-cortin-like kinase 1)	F: TAAGGCGCAGAGATACAGCA<br>R: GGTTCGGTAGAAGCTGCAAT	85
**Housekeeping genes:**		
GADPH (Glyceraldehyde-3-phosphate dehydrogenase)	F: ACCCAGAAGACTGTGGATGG<br>R: AAGCAGGGATGATGTTCTGG	79
RPL13A (Ribosomal protein L13A)	F: ATTGTGGCCAAGCAGGTACT<br>R: AATTGCCAGAAATGTTGATGC	76
PPIA (Peptidylprolyl isomerase A)	F: CAAGACTGAGTGGTTGGATGG<br>R: TGTCCACAGTCAGCAATGGT	138
