# Supplementary material for: Mucosal Barrier and Th2 Immune Responses Are Enhanced by Dietary Inulin in Pigs Infected With Trichuris suis
Source: Front Immunol. 2018 Nov 9;9:2557. doi: 10.3389/fimmu.2018.02557 (PMC6237860; doi:10.3389/fimmu.2018.02557)
Supplement: Supplementary file 4 [file Data_Sheet_4.PDF]

```
title = MykParLab, 16S RDP
author = SSI
service site = SSI
primer-file = Lee_16S.primers
sample-file = samples.txt
reference-data = RDP_SSU_340-807- All keep-all-files = yes
zip-outputs = no
allow-missing-inputs = yes
allow-missing-outputs = no
quality-type = Sanger

# ---------------------------------------------------------------------------
# EXTRACTION
# ---------------------------------------------------------------------------
title = Sequence extraction
trim-end-strictness = 95
pair-regex-forward = _R1_
pair-regex-reverse = _R2_
extract-reverse = no
write-failed = no

# ------------------------------------------------------------------------
# QUALITY TRIM
# ------------------------------------------------------------------------
title = Start quality trim
 window-length = 15
window-match = 14
minimum-quality = 99%

title = End quality trim
window-length = 30
window-match = 28
minimum-quality = 99%

# ------------------------------------------------------------------------
# LOW COMPLEXITY FILTER
# ------------------------------------------------------------------------
title = Low complexity filter
pattern-string-nomatch = p1=2...2 p1 p1 p1 p1 p1 p1 p1 p1 p1
match-forward = yes

# ---------------------------------------------------------------------------
# JOIN PAIR MATES
# ---------------------------------------------------------------------------
title = Pair mate joining
forward-filter = .F.
reverse-filter = .R.
complement-reverse = no
minimum-similarity = 90
minimum-overlap = 18
include-unjoined = yes
include-singlets = no

# ----------------------------------------------------------------------------
# LENGTH AND QUALITY FILTER
# ----------------------------------------------------------------------------
title = Length and quality filtering
title = Length filter minimum-length = 250
title = Quality filter minimum-quality = 99 minimum-strict = 95

# ----------------------------------------------------------------------------
# DE-REPLICATION
# ----------------------------------------------------------------------------
title = Sequence uniqification

# ----------------------------------------------------------------------------
# CHIMERA CHECK
# ----------------------------------------------------------------------------
title = Chimera filtering reference-data = RDP_SSU_340-807-Chim
pre-clustering = 96%
minimum-score = 15
```

**Figure S2: Workflow for read assembly and taxa annotation done by BIONmeta**

```
# ---------------------------------------------------------------------------
# SAMPLE CLUSTERING
# ---------------------------------------------------------------------------
title = Sample sequence clustering
minimum-oligo-similarity = 96%
minimum-sequence-length = 300
minimum-base-quality = 0%

# ---------------------------------------------------------------------------
# REFERENCE SIMILARITIES
# ---------------------------------------------------------------------------
title = RDP similarities
 top-similarity-range = 5
minimum-base-quality = 0%
minimum-oligo-similarity = 60%

# ---------------------------------------------------------------------------
# REFERENCE PROFILING
# ---------------------------------------------------------------------------
title = Similarities profiling
input-step = sequence-similarities-simrank
minimum-oligo-similarity = 60%
maximum-low-quality = 100%
minimum-read-sum = 1
prefer-named-taxa = yes
prefer-named-favorites = yes
minimum-favorite-score = 10%
minimum-favorite-similarity = 85%
minimum-favorite-readsum = 1
single-favorite-map = no
with-self-clusters = yes
minimum-self-similarity = 60
with-debug-dumps = no
sequence-link-ranks = family, genus, species
cumulative-links = no

# # title = Taxonomy tables, favorites only
# input-step = organism-taxonomy-profiler
# minimum-row-max-percent = 0%
# minimum-row-value-percent = 0%
# favorites-only = yes
# normalized-column-total = 100000
# with-self-clusters = no
# title = Taxonomy tables, with non-favs.

input-step = organism-taxonomy-profiler
minimum-row-max-percent = 0%
minimum-row-value-percent = 0%
with-ambiguous-taxa = yes
favorites-only = no
normalized-column-total = 100000
with-self-clusters = no

# # title = Taxonomy tables, favs only + self
# input-step = organism-taxonomy-profiler
# minimum-row-max-percent = 0%
# minimum-row-value-percent = 0%
# favorites-only = yes
# normalized-column-total = 100000
# with-self-clusters = yes

# title = Taxonomy tables, non-favs + self
input-step = organism-taxonomy-profiler
minimum-row-max-percent = 0%
minimum-row-value-percent = 0%
with-ambiguous-taxa = yes
favorites-only = no
normalized-column-total = 100000
with-self-clusters = yes
```

**Figure S2 continued: Workflow for read assembly and taxa annotation done by BIONmeta**
