# Supplementary material for: Mucosal Barrier and Th2 Immune Responses Are Enhanced by Dietary Inulin in Pigs Infected With Trichuris suis
Source: Front Immunol. 2018 Nov 9;9:2557. doi: 10.3389/fimmu.2018.02557 (PMC6237860; doi:10.3389/fimmu.2018.02557)
Supplement: Supplementary file 5 [file Data_Sheet_5.PDF]

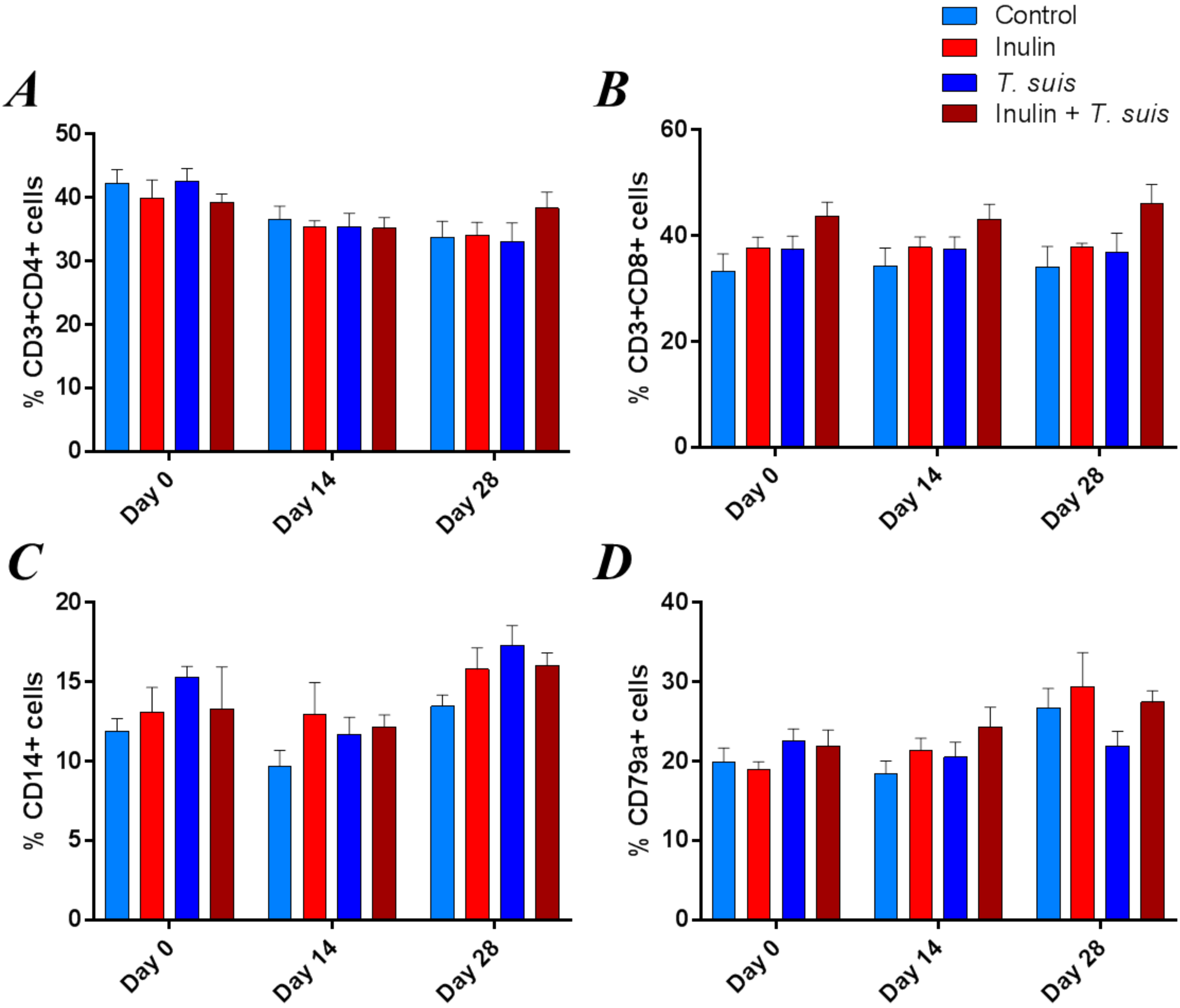

Figure S3: Flow cytometric analysis of peripheral blood mononuclear cells (PBMCs) at day 0, day 14 and day 28 post infection. Percentage of **(A)** CD3+CD4+ T helper cells, **(B)** CD3+CD8+ cytotoxic T cells, **(C)** CD14+ monocytes, **(D)** CD79a+ B cells. Data are presented as means and error bars represent SEM. No significant influence of diet nor infection was observed.
