# Supplementary material for: Mucosal Barrier and Th2 Immune Responses Are Enhanced by Dietary Inulin in Pigs Infected With Trichuris suis
Source: Front Immunol. 2018 Nov 9;9:2557. doi: 10.3389/fimmu.2018.02557 (PMC6237860; doi:10.3389/fimmu.2018.02557)
Supplement: Supplementary file 6 [file Data_Sheet_6.PDF]

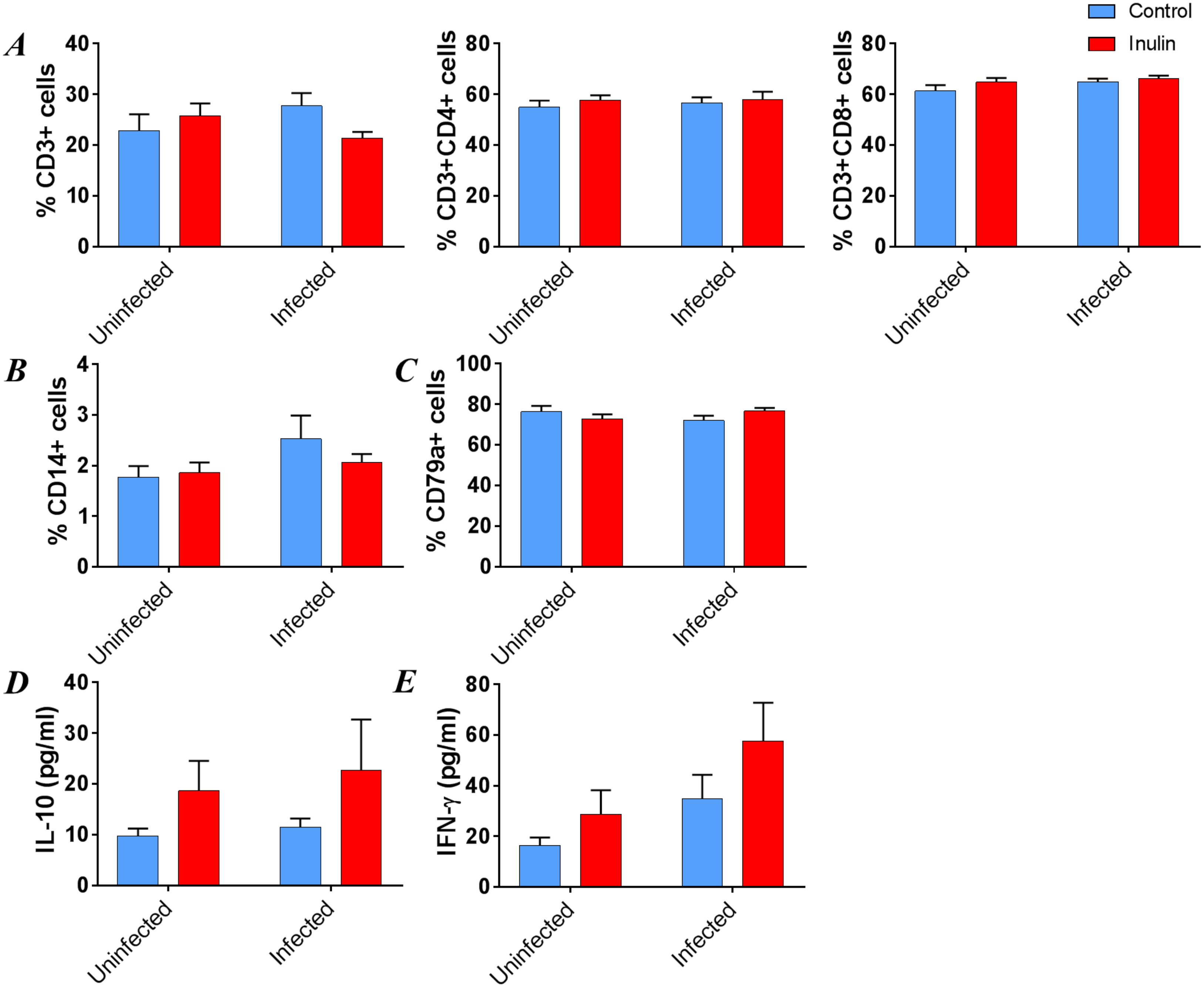


**Figure S4:** Flow cytometric analysis of ileo-caecal lymph node (CLN) cells isolated at day 28 post infection. **(A)** Percentage of CD3+ T cells, CD3+CD4+ helper T cells, and CD3+CD8+ cytotoxic T cells. **(B)** Percentage of CD14+ monocytes. **(C)** Percentage of CD79a+ B cells. **(D)** Cytokines IL-10, and **(E)** IFN-γ secreted from cultured CLN cells after 24 hours stimulation with PHA mitogen. Data are presented as means and error bars represent SEM. No significant influence of diet nor infection was observed.
