# Supplementary material for: Mucosal Barrier and Th2 Immune Responses Are Enhanced by Dietary Inulin in Pigs Infected With Trichuris suis
Source: Front Immunol. 2018 Nov 9;9:2557. doi: 10.3389/fimmu.2018.02557 (PMC6237860; doi:10.3389/fimmu.2018.02557)
Supplement: Supplementary file 7 [file Data_Sheet_7.PDF]

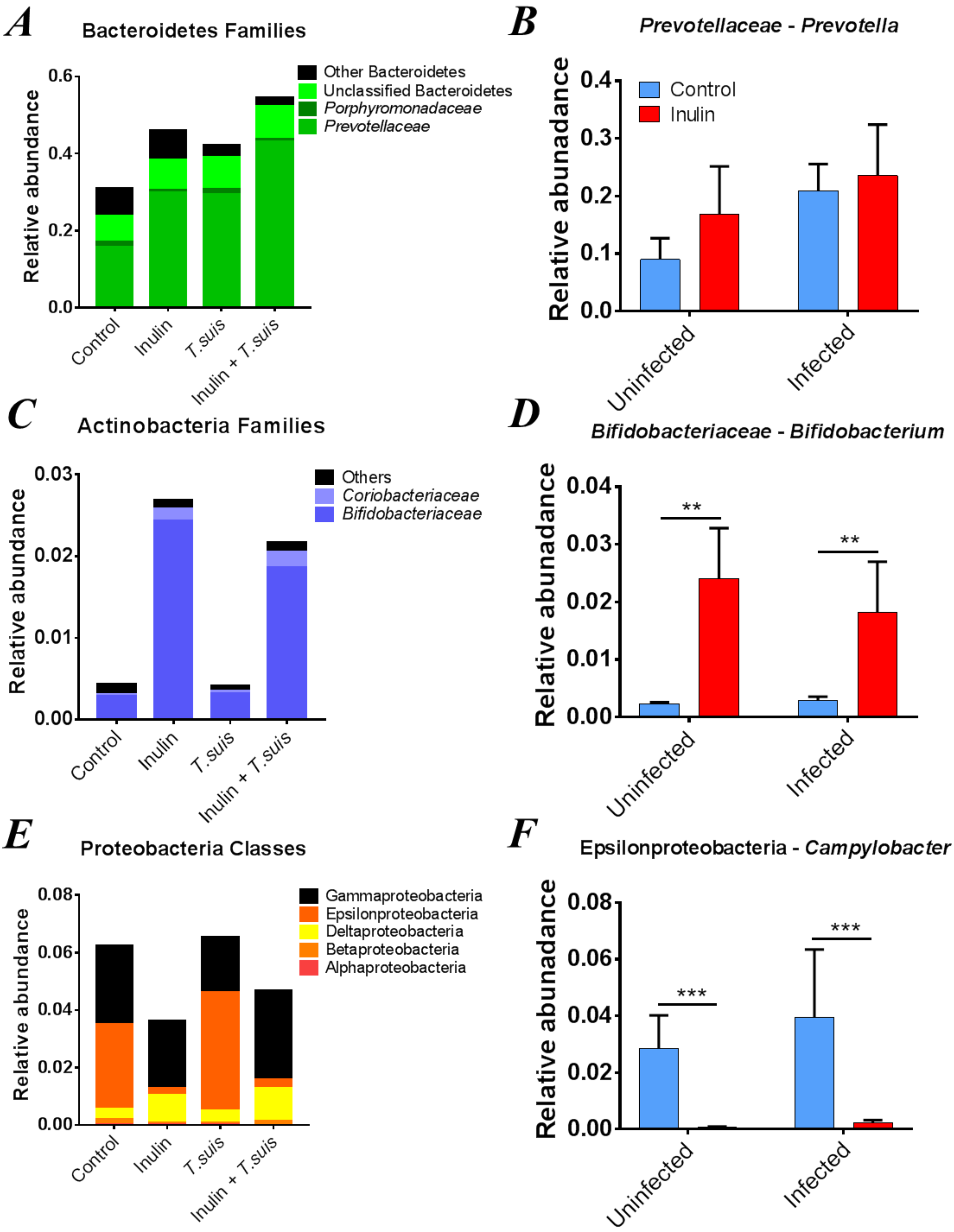

Figure S5: Intestinal microbiota abundance: Bacteroidetes, Actinobacteria and Proteobacteria. Relative distribution of selected taxa: **(A)** Families belonging to the Bacteroidetes phylum, where *Prevotellaceae* is the most abundant; **(B)** *Prevotella* relative abundance; **(C)** Families belonging to the Actinobacteria phylum, where *Bifidobacteriaceae* is the most abundant; **(D)** *Bifidobacterium* relative abundance; **(E)** Classes of Proteobacteria, where a decrease in Epsilonproteobacteria is seen for inulin-fed animals; **(F)** *Campylobacter* relative abundance. Data are presented as means and error bars represent SEM (**$p \leq 0.01$, ***$p \leq 0.005$, by mixed model).
