# Supplementary material for: Mucosal Barrier and Th2 Immune Responses Are Enhanced by Dietary Inulin in Pigs Infected With Trichuris suis
Source: Front Immunol. 2018 Nov 9;9:2557. doi: 10.3389/fimmu.2018.02557 (PMC6237860; doi:10.3389/fimmu.2018.02557)
Supplement: Supplementary file 8 [file Data_Sheet_8.PDF]

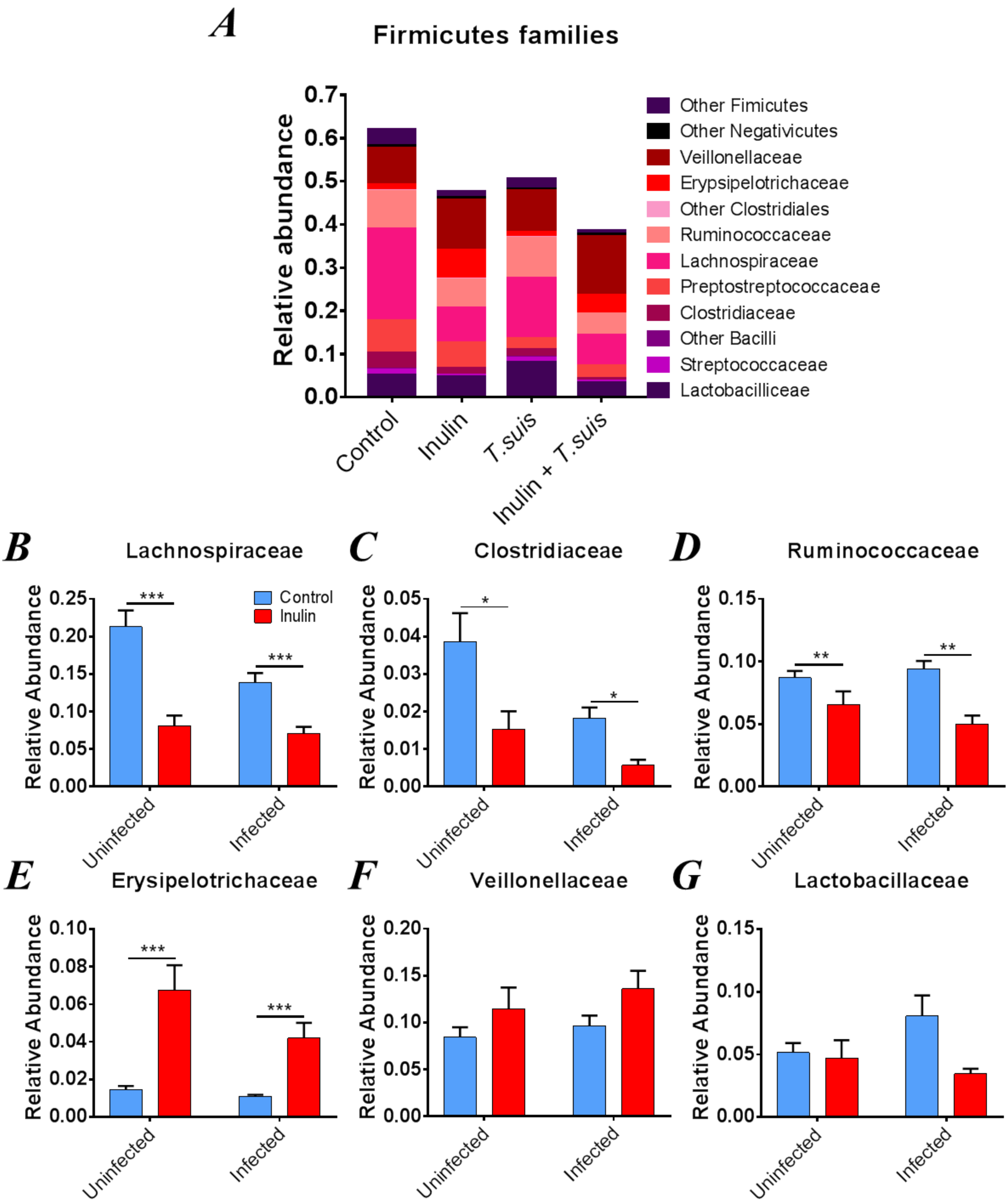

Figure S6: Intestinal microbiota abundance: Firmicutes. Relative distribution of families belonging to the Firmicutes phylum for each treatment group **(A)**. Selected significantly altered families include: **(B)** Lachnospiraceae; **(C)** Clostridiaceae; **(D)** Ruminococcaceae; **(E)** Erysipelotrichaceae; **(F)** Veillonellaceae; **(G)** Lactobacillaceae. Data are presented as means and error bars represent SEM (*$p \leq 0.05$, **$p \leq 0.01$, ***$p \leq 0.005$, by mixed model).
