# Supplementary material for: Mucosal Barrier and Th2 Immune Responses Are Enhanced by Dietary Inulin in Pigs Infected With Trichuris suis
Source: Front Immunol. 2018 Nov 9;9:2557. doi: 10.3389/fimmu.2018.02557 (PMC6237860; doi:10.3389/fimmu.2018.02557)
Supplement: Supplementary file 9 [file Data_Sheet_9.PDF]

Table S3: Relative expression data of all statistically significant immune genes from proximal colon tissue ($p \leq 0.05$). * Granzyme A (GZMA) relative expression levels divided by 1000. # = $p < 0.1$, indicates trend towards statistical significance, determined by mixed model.

Immune function	Immune gene	Relative expression				Significance (*p*-value)	
		Control	*T. suis*	Inulin	Inulin + *T. suis*	Diet	Infection
Th1	*IL1A*	2.6	3.9	1.5	1.8	0.000	0.013
	IL1B	8.3	6.8	2.9	3.0	0.007	
	IL6	3.4	6.0	2.1	4.5		0.031
	IL8	4.1	3.6	2.4	2.0	0.007	
	IL15 #	8.7	5.3	10.0	8.5		0.095
	IFNG	11.3	4.9	8.7	2.6	0.017	0.000
	TNF	7.2	4.1	5.5	3.4		0.051
	TNFRSF4	5.3	3.8	2.0	2.3	0.001	
	TLR3	2.1	1.6	2.7	1.9	0.004	0.000
	TLR4	2.3	2.2	2.4	1.7		0.033
	TLR7	5.7	2.8	5.0	2.9		0.006
	TLR8	5.2	2.6	3.9	1.8		0.004
	CD40 #	13.3	6.6	6.0	7.4	0.063	
	CD86	3.9	2.2	3.6	1.5		0.000
	CD163	4.9	3.0	4.2	2.4		0.001
	PRF1	2.0	1.7	2.4	1.4		0.022
	*GZMA**	11.7	5.8	17.1	8.7		0.011
	GZMB	2.5	1.6	2.9	1.5		0.017
	KLRK	4.4	2.7	5.5	2.8		0.002
	CCL3	3.0	2.0	3.2	1.9		0.015
	CXCL9	36.6	7.6	19.5	4.4	0.027	0.000
Th2	*IL5*	1.9	2.2	3.2	2.3	0.026	
	IL13	2.0	4.6	4.4	8.6	0.001	0.002
	ARG1 #	6.0	15.1	3.1	10.0		0.081
	CCL17	2.8	5.0	2.8	5.7		0.000
	CCL22	3.5	4.5	1.7	3.8	0.065	0.023
T-reg	*IL10*	9.7	5.3	5.9	3.2	0.013	0.002
	TGFB1	11.8	4.3	5.7	3.1	0.043	0.020
Mucosal barrier function	*SLC2A5*	27.8	50.1	104.4	53.9	0.006	
	SLC5A1	2.2	3.4	1.8	2.5	0.000	0.000
	MUC2 #	6.5	12.2	8.4	16.0		0.078
	MUC5AC	11.3	156.6	4.3	57.2		0.006
	TFF2	4.9	28.0	4.5	38.7		0.006
	TFF3	1.3	2.4	2.1	3.3	0.000	0.000
	RETNLB	1.5	20.7	2.3	40.1		0.005
	SOX9 #	1.8	1.9	2.4	2.5	0.074	
	DCLK1	3.7	2.4	4.9	5.5	0.003	
